# Endoplasmic Reticulum Stress Delays Choroid Development in the *HCAR1* Knockout Mouse

**DOI:** 10.1016/j.ajpath.2024.09.002

**Published:** 2024-09-26

**Authors:** Monir Modaresinejad, Xiaojuan Yang, Mohammad A. Mohammad Nezhady, Tang Zhu, Emmanuel Bajon, Xin Hou, Houda Tahiri, Pierre Hardy, José C. Rivera, Pierre Lachapelle, Sylvain Chemtob

**Affiliations:** ∗ Program in Biomedical Science, Faculty of Medicine, Université de Montreal, Montreal, Quebec, Canada; ¶ Program in Molecular Biology, Faculty of Medicine, Université de Montréal, Montreal, Quebec, Canada; ‡ School of Optometry, Université de Montréal, Montreal, Quebec, Canada; † Department of Pediatrics, Ophthalmology and Pharmacology, Centre de Recherche du CHU Sainte-Justine, Montréal, Quebec, Canada; § Departments of Ophthalmology and Neurology-Neurosurgery, Research Institute of the McGill University Health Centre–Montreal Children's Hospital, Montreal, Quebec, Canada

## Abstract

The subretina, composed of the choroid and the retinal pigment epithelium (RPE), plays a critical role in proper vision. In addition to phagocytosis of photoreceptor debris, the RPE shuttles oxygen and nutrients to the neuroretina. For their own energy production, RPE cells mainly rely on lactate, a major by-product of glycolysis. Lactate, in turn, conveys most of its biological effects via the hydroxycarboxylic acid receptor 1 (HCAR1). Herein, the lactate-specific receptor, HCAR1, was found to be exclusively expressed in the RPE cells within the subretina, and *Hcar1*^*−/−*^ mice exhibited a substantially thinner choroidal vasculature during development. Notably, the angiogenic properties of lactate on the choroid were impacted by the absence of *Hcar1*. HCAR1-deficient mice exhibited elevated endoplasmic reticulum stress along with eukaryotic translation initiation factor 2α phosphorylation, a significant decrease in the global protein translation rate, and a lower proliferation rate of choroidal vasculature. Strikingly, inhibition of the integrated stress response using an inhibitor that reverses the effect of eukaryotic translation initiation factor 2α phosphorylation restored protein translation and rescued choroidal thinning. These results provide evidence that lactate signalling via HCAR1 is important for choroidal development/angiogenesis and highlight the importance of this receptor in establishing mature vision.

## Graphical abstract

**Figure:**
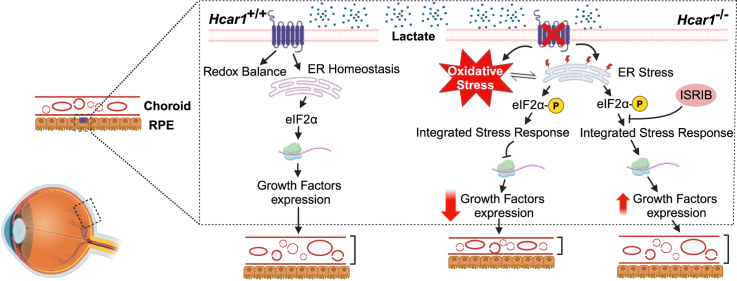

The choroid is a highly vascularized structure of the subretina^1^ that supplies O_2_ and nutrients to the retinal pigment epithelium (RPE) and photoreceptors. It is primarily a vascular tissue overlaying the RPE and outer retina, contributing to ocular growth and development.^2^ The underlying RPE is a monolayer of post-mitotic cells that assists in the critical recycling of photoreceptor outer segments, acts as a barrier to control the passage of fluids and nutrients to photoreceptors, maintains the integrity of the choroidal vasculature, and protects the retina.^3^^,^^4^ In this context, RPE cells also have a role in the secretion of a variety of growth and immunosuppressive factors to maintain choriocapillaris well-being.^4^^,^^5^

The retina is the most oxygen-consuming tissue in the body and, hence, metabolic disturbances are key contributors to ocular pathology.^6^ Indeed, photoreceptor activity requires a high metabolic activity.^7^ RPE cells phagocytose photoreceptor debris and absorb UV light; both functions lead to high reactive oxygen species (ROS) accumulation and, thus, require exquisite oxidation-reduction balance management.^8^ Cell stress resulting from ROS accumulation causes RPE dysfunction, in turn leading to visual impairment.^9^ Primary malfunctions of RPE can aggravate visual cell loss and lead to blindness.^10^ The RPE and the neuroretina are both derived from embryonic neuroepithelial tissue and codifferentiated during the development of the optic cup.^11^ The integrity of the choroid (and retina) is partly dependent on the generation of major RPE-derived growth factors, such as insulin-like growth factor 1 (IGF1), transforming growth factor beta 1 (TGFB1), fibroblast growth factor 2 (FGF2), or vascular endothelial growth factor A (VEGFA).^4^ Hence, the growth and function of the highly vascularized choroid is, to a large extent, governed by the RPE.^1^

In the subretina, composed of the RPE and the choroid, nearly 80% of glucose is converted into lactate, compared with 20% conversion in the inner retina.^12^ RPE cells use lactate as fuel for ATP production via oxidative phosphorylation. This is presumed to recycle lactate and permit higher glucose availability for the photoreceptors.^13^ Strikingly, metabolites such as succinate and lactate are determinants of retinal vascular development.^14^^,^^15^ As the endogenous ligand of the G-protein–coupled receptor hydroxycarboxylic acid receptor 1 (HCAR1), also known as GPR81, lactate is an important signaling molecule.^16^ More specifically, lactate-HCAR1 signaling plays a role in several physiological and pathologic processes relevant to the eye, including lipid metabolism,^17^^,^^18^ inflammatory response,^19^^,^^20^ glucose homeostasis,^16^ angiogenesis,^15^^,^^21^ and cancer.^22^

On the basis of previous findings that HCAR1 regulates inner neuroretinal vascular development,^15^ it was hypothesized that ontogenic changes in the lactate receptor would also control vascular development in the subretina (choroid). The abundant metabolic generation of lactate in the subretina along with the critical role of the RPE in sustaining the choroid led us to explore the role of the lactate receptor, HCAR1, during choroidal development using the *Hcar1*^*−/−*^ mouse model. Further dissection in *ex vivo* and *in vivo* experiments revealed the underlying molecular mechanisms affected by HCAR1 in relation to choroidal integrity.

## Materials and Methods

### Animals

C57BL/6 mice were obtained from the Jackson Laboratory (Bar Harbor, ME). *Hcar1* knockout (KO) mice were purchased from Lexicon Pharmaceuticals (The Woodlands, TX). In *Hcar1-*KO mice, the transmembrane domain 2 of the coding region of *Hcar1* (100 bp) is replaced by a 4-kb IRES-lacZ-neo cassette in C57BL/6J mice.^20^ Mice were maintained on standard environmentally controlled conditions (temperature: 20°C ± 2°C, humidity: 60% ± 5%, 12 hours dark/12 hours light cycle). Food and water were taken *ad libitum.* Mice were examined at different post-natal times (PTs), including PT7, PT9, PT12, and PT15.

Integrated stress response inhibitor (ISRIB) treatment was done by injection of 200 nmol/L of ISRIB or dimethyl sulfoxide as a vehicle intravitreally in PT7, PT9, and PT12 mice. Then, the mice were sacrificed at PT9, PT12, and PT15, respectively, and eyes were enucleated for further experiments.

### Ethics

All the experiments were performed in compliance with the Association for Research in Vision and Ophthalmology statement for the Use of Animals in Ophthalmic and Vision Research and approved by the Animal Care Committee of the Sainte-Justine Hospital according to guidelines established by the Canadian Council on Animal Care.

### Chemical Preparations

l-lactate (L7022; Sigma-Aldrich, St. Louis, Mo) was dissolved in phosphate-buffered saline (PBS), and the pH of the solution was adjusted with NaOH solutions (final pH 6.5 to 7.3; final concentration, 10 mmol/L). 3,5-Dihydroxybenzoic acid (D110000; Sigma-Aldrich; final concentration, 80 μmol/L) was dissolved in PBS as well. ISRIB (SML0843; Sigma-Aldrich) was dissolved in dimethyl sulfoxide (final concentration, 200 nmol/L).

### RPE/Choroid Complex Collection

Eyes were enucleated and kept in cold PBS. Then, eyes were opened from the ora serrata to remove the anterior part (cornea and lens). The optic nerve was cut, and the retina was separated from the posterior eye cup. For cell isolation, sterile procedures were performed. The obtained RPE/choroid complex was then dried briefly on delicate wipes followed by rapid freezing on dry ice if applicable. Eyes from a cohort of mice were prepared for experiments, including RNA and protein extraction, growth factor microarray, ROS/reactive nitrogen species (RNS) detection, and superoxide dismutase activity.

### RNA Extraction and Quantitative RT-PCR

Total RNA was extracted from RPE/choroid complex and primary RPE cells using an RNA extraction kit (RNeasy plus minikit; Qiagen, Manchester, UK). The RNA concentration and integrity were determined with NanoDrop 1000 spectrophotometer (Thermo Fisher Scientific, Wilmington, DE). Reverse transcription was performed using iScript cDNA SuperMix (Bio-Rad Laboratories, Hercules, CA). Primers (forward and reverse primer sequences) were designed by the Primer-Blast program and were purchased from Alpha DNA (Montreal, QC, Canada) or Integrated DNA Technologies (Table 1). SYBR Green Master mix kit (Bio-Rad) and Stratagene Mx3000p (Stratagene, San Diego, CA) detection system were used to perform quantitative analysis of gene expression. Quantitative RT-PCR assays were analyzed on triplicate samples, and their C_T_ numbers were averaged. Gene expressions were normalized to 18S expression (18S universal primer; Bio-Rad) using the ΔC_T_ method, and the fold change expression was analyzed by the ΔΔC_T_ method.

**Table 1:** Mouse Primers Used

Target gene	Forward primer	Reverse primer
*Hcar1*	5′-CATCTTGTTCTGCTCGGTCA-3′	5′-GAGGAAGTAGAGCCTAGCCA-3′
*Il1b*	5′-GCCCATCCTCTGTGACTCAT-3′	5′-AGGCCACAGGTATTTTGTCG-3′
*Tnf*	5′-TTCGAGTGACAAGCCTGTAG-3′	5′-GACGGCAGAGAGGAGGTTGAC-3′
*Cxcl15* (*Il8* homolog)	5′-CCGTCCCTGTGACACTCAAG-3′	5′-ACAGAAGCTTCATTGCCGGT-3′
*COX2*	5′-GCTGCGGAAGAAGGCTTTTG-3′	5′-GGTTGGGTCCCAGGAATGAG-3′
*Nlrp3*	5′-AGCCTTCCAGGATCCTCTTC-3′	5′-CTTGGGCAGCAGTTTCTTTC-3′
*Pla2*	5′-TCTTGATCCCCAATGCTTCG-3′	5′-GCTGCATCTCCTTTTTGCTG-3′
*Ccl2*	5′-GCTCAGCCAGATGCAGTTA-3′	5′-TGTCTGGACCCATTCCTTCT-3′
*Mmp3*	5′-ACTCTACCACTCAGCCAAGG-3′	5′-TCCAGAGAGTTAGACTTGGTGG-3′
*Il1rn*	5′-CTGCACTTCCACAGTCCAGA-3′	5′-ATATGTGATGCCCTGGTGGT-3′
*Il10*	5′-TGAATTCCCTGGGTGAGAAG-3′	5′-ACACCTTGGTCTTGGAGCTT-3′
*Vegfa*	5′-GCCCTGAGTCAAGAGGACAG-3′	5′-CTCCTAGGCCCCTCAGAAGT-3′
*Fgf2*	5′-TGGCTTCTAAGTGTGTTACAG-3′	5′-GTTTCAGTGCCACATACCAAC-3′
*Angpt2* (*Ang2*)	5′-ATGAGCCCAGGTCCTTTGTT-3′	5′-AGAGGTTAGCTTTCTTTTCACCA-3′
*Serpinf1*	5′-GGGGCCCTCCTCTTCATAG-3′	5′-GAGCCCGTTCATGTTACAAGT-3′
*ApoE*	5′-TTCGGAAGGAGCTGACTGG-3′	5′-TTCTTGTGTGACTTGGGAGC-3′
*Sema3f*	5′-TCGCGCACAGGATTACATCTT-3′	5′-ACCGGGAGTTGTACTGATCTG-3′
*TSP1*	5′-CAAAGCCTGCAAGAAAGACG-3′	5′-TTGGACAGGCATCCATCAAT-3′
*Igf1r*	5′-GCTTCTGTGAACCCCGAGTATTT-3′	5′-TGGTGATCTTCTCTCGAGCTACCT-3′
*Igfbp2*	5′-GCAGACCTCGGGTGAGAAAA-3′	5′-TCGTCATCACTGTCTCCCAAC-3′
*Vegfr2* (*Kdr*)	5′-TTTGGCAAATACAACCCTTCAGA-3′	5′-GCAGAAGATACTGTCACCACC-3′
*Bcl2*	5′-TGTGGCCTTCTTTGAGTTCG-3′	5′-TTCCACAAAGGCATCCCAG-3′
*Bad*	5′-CCAGTGATCTTCTGCTCCACATCCC-3′	5′-CAACTTAGCACAGGCACCCGAGGG-3′
*Casp3*	5′-ATGGGAGCAAGTCAGTGGAC-3′	5′-CGTACCAGAGCGAGATGACA-3′
*Fasl*	5′-TGGCTACCGGTGGTATTTTT-3′	5′-GGGTTGGCTATTTGCTTTTC-3′
*Ripk3*	5′-AAGTGCAGATTGGGAACTACAACTC-3′	5′-AGAATGTTGTGAGCTTCAGGAAGTG-3′
*Asns*	5′-GAGAAACTCTTCCCAGGCTTTG-3′	5′-CAAGCGTTTCTTGATAGCGTTGT-3′
*Trib3*	5′-TGTCTTCAGCAACTGTGAGAGGA-3′	5′-CAGTCATCACGCAGGCATC-3′
*Ddit3 (Chop)*	5′-CGGAACCTGAGGAGAGAGTG-3′	5′-CGTTTCCTGGGGATGAGATA-3′

### Immunohistofluorescence

Eyes or primary RPE cultures after enucleation were fixed in 4% paraformaldehyde for 1 to 1.5 hours or 10 minutes, respectively. Eye cups were further adequately dehydrated by 30% sucrose overnight at 4°C and subsequently embedded in OCT (Thermo Fisher Scientific, Waltham, MA) medium; finally, they were frozen on dry ice and kept at –80°C. Sagittal sections (12 μm thick) were prepared (model CM3050S cryostat; Leica Microsystems, Wetzlar, Germany) and kept at –80°C until further processing. Eye cryosections or slides of RPE cells were then blocked with 1% bovine serum albumin and 0.1% Triton X-100 (T-8787; Sigma-Aldrich, St. Louis, MO) in PBS and were afterward incubated overnight with primary antibodies (Table 2) followed by a secondary antibody incubation for at least 1 hour at room temperatures. Nuclei were stained with DAPI (Sigma-Aldrich), and samples were mounted by using a gold antifade medium (P36961; Thermo Fisher Scientific). Slides were then analyzed by epifluorescent microscopy (E800; Nikon Eclipse, Melville, NY) or confocal microscope (SP8, Leica, Germany).

**Table 2:** Antibodies and Reagents Used

No.	Name	Catalog no.	Concentration/dilution	Company
1	Anti-mouse RPE65 antibody	Sc-390787	1:250	Santa Cruz Biotechnology (Dallas, TX)
2	Anti-rabbit HCAR1 antibody	SAB1300790	1:400	Sigma-Aldrich (St. Louis, MO)
3	Rhodamine-conjugated *Griffonia simplicifolia* lectin I (GSL-1)	RL-1102	1:500	Vector Laboratories (Newark, CA)
4	FITC-phalloidin	00030	1:400	Biotium (San Francisco, CA)
5	Anti-rabbit Ki-67 antibody	Ab16667	1:200	Abcam (Boston, MA)
6	Anti-mouse eIF2α (D-3) antibody	Sc-133132	1:100	Santa Cruz Biotechnology
7	Anti-rabbit pho-eIF2α antibody	Ab32157	1:500	Abcam
8	Anti-mouse BiP antibody	BD 610978	1:1000	BD Bioscience (Franklin Lakes, NJ)
9	Anti-mouse PDI antibody	Ab2792	1:1000	Abcam
10	Anti-mouse HSP90 antibody	Sc-13119	1:1000	Santa Cruz Biotechnology
11	Anti-rabbit phospho-PERK antibody	MA5-15033	1:1000	Invitrogen (Waltham, MA)
12	Anti-rabbit PERK antibody	BS-2469R	1:1000	Bioss (Woburn, MA)
13	Anti-rabbit ATF4/creb-2 (b3) antibody	Sc-390063	1:100	Santa Cruz Biotechnology
14	Anti-rabbit β-tubulin antibody	10068-1-AP	1:1000	Proteintech (Chicago, IL)
15	Anti-mouse β-actin (C4) antibody	Sc-47778	1:1000	Santa Cruz Biotechnology
16	Anti-rabbit NRF2 antibody	Ab31163	1:500	Abcam
17	Anti-rabbit phospho-IRE-1α antibody	S724	1:500	Sigma-Aldrich
18	Anti-rabbit IRE-1α antibody	3294	1:500	Cell Signaling Technology (Danvers, MA)
19	Anti-mouse XBP-1s antibody	40435 (E9V3E)	1:1000	Cell Signaling Technology
20	Anti-rabbit ATF-6 antibody	EPR22690-84	1:1000	Abcam
21	Anti-rabbit Iba-1 antibody	Ab178847	1:500	Abcam
22	Anti-mouse podocalyxin antibody	MAB1556	1:250	R & D Biosystems (Minneapolis, MN)
23	α-SMA antibody	A 2547	1:500	Thermo Fisher Scientific (Waltham, MA)
24	Secondary anti-rabbit HRP-linked antibody	7074S	1:1000	Cell Signaling Technology
25	Secondary anti-mouse HRP-linked antibody	172-1011	1:1000	Bio-Rad (Hercules, CA)
26	l-lactate	L7022	10 mmol/L in PBS	Sigma-Aldrich
27	DHBA	D110000	80 μmol/L in PBS	Sigma-Aldrich
28	ISRIB	SML0843	200 nmol/L in DMSO	Sigma-Aldrich
29	DMEM			Invitrogen (Waltham, MA)
30	Fungizone	15290026		Thermo Fisher Scientific
31	Trypsin	T9201		Sigma-Aldrich
32	Collagenase I	C0130		Sigma-Aldrich
33	Attachment factor	S006100		Gibco, Fisher Scientific (Waltham, MA)
34	iScript cDNA SuperMix			Bio-Rad
35	RNA extraction kit (RNeasy plus minikit)	74136		Qiagen (Manchester, UK)
36	SYBR Green Master mix kit	1725124		Bio-Rad
37	18S universal primer			Bio-Rad
38	TritonX-100	T-8787	0.1% in PBS	Sigma-Aldrich
39	Gold antifade medium	P36961		Thermo Fisher Scientific
40	DAPI	D9542		Sigma-Aldrich
41	Matrigel			Corning Life Science (Tewksbury, MA)
42	EBM2-Media	CC-3156		Lonza (Walkersville, MD)
43	Dispase II	C756V28		Roche (Indianapolis, IN)
44	Hanks’ balanced salt solution	88284		Invitrogen
45	RIPA buffer	9806		Cell Signaling Technology
46	Proteinase inhibitor cocktail	11697498001		Roche
47	Bradford	5000006		Bio-Rad
48	Western Lightning Plus-ECL	NEL103E001 EA		PerkinElmer (Waltham, MA)
49	Mouse growth factor array C3			RayBiotech (Peachtree Corners, GA)
50	OxiSelect *In Vitro* ROS/RNS Assay Kit	STA-347-5		Cell Biolabs (San Diego, CA)
51	SOD Assay Kit	19160		Sigma-Aldrich
52	*In Situ* Cell Death Detection Kit (TUNEL assay)	C755B40		Roche
53	Click-&-Go Plus 488 OPP Protein Synthesis Assay Kit	1493		Click-Chemistry Tool (Scottsdale, AZ)
54	O.C.T			Thermo Fisher Scientific
55	H_2_O_2_	349887		Sigma-Aldrich

α-SMA, α-smooth muscle actin; ATF, activating transcription factor; BiP, binding immunoglobulin protein; DHBA, 3, 5-dihydroxybenzoic acid; DMEM, Dulbecco’s modified Eagle’s medium; DMSO, dimethyl sulfoxide; eIF2α, eukaryotic translation initiation factor 2α; FITC, fluorescein isothiocyanate; HRP, horseradish peroxidase; HSP, heat shock protein; Iba-1, ionized calcium-binding adaptor molecule 1; IRE-1, inositol-requiring enzyme-1; ISRIB, integrated stress response inhibitor; NRF2, nuclear factor erythroid 2-related factor 2; PBS, phosphate-buffered saline; PDI, protein disulfide isomerase; PERK, protein kinase RNA-like ER kinase; XBP-1s, spliced X-box binding protein 1.

### Bleaching of Choroidoscleral Tissue

Labeling choroidoscleral tissue requires an extra step. After tissue fixation in 4% paraformaldehyde for 1 hour, fixed choroidoscleral tissues were washed with PBS (three times, 5 minutes), and then for bleaching, 1% H_2_O_2_ was used (in 1× PBS; 349,887; Sigma-Aldrich) to preserve tissue architecture and the natural epitope. Tissues were submerged in 1% H_2_O_2_ and kept at 55°C for 35 minutes. Once the tissue was reasonably clear, the bleaching process was finished. Then, the tissues were washed with PBS (three times, 5 minutes) and used for the immunohistofluorescence staining to visualize the choroidoscleral tissue.^23^

### Quantification of Choroidal Thickness

Rhodamine-conjugated *Griffonia simplicifolia* lectin I (1:500; Vector Laboratories, Newark, CA) staining was used for visualizing vasculature and to designate the choroidal endothelium. Sections that contained the optic nerve head were subjected to this experiment. The choroidal vasculature thickness was measured on digital images taken by confocal microscopy under ×40 amplifications using ImageJ version 1.53 t software (NIH, Bethesda, MD; *https://imagej.net/ij*). Choroidal thickness was evaluated at 150-μm intervals throughout the choroid, starting with the optic nerve (the 0-μm position). The results were averaged and displayed into a spider graph.^24^

### Choroidal Sprout Assay

The 4-week–old mice were used for choroidal explant assay, according to the method that was previously described.^25^ Eye enucleations were completed under aseptic conditions and maintained in EBM-2 media (CC-3156; Lonza, Walkersville, MD). All connective tissue was removed from the external part of the eyes and then the cornea, lens, retina, and optic nerve head were removed from the eye cup by an incision under the ora serrata. RPE/choroid complex was further cut into 1- to 2-mm sections and placed in 30 μL Matrigel (Corning Life Science, Tewksbury, MA) in a 24-well plate. After solidification of Matrigel in the incubator for 30 minutes, choroidal explants were kept in EGM-2 media. All these procedures were done under a laminar hood to maintain sterile conditions. Choroidal complex alone (without the RPE cell layer) was prepared on the basis of the previous method.^26^ Overall, the procedure for the choroidal sprout assay was like the preparation of the RPE/choroid complex except for the digestion step. After removing connective tissue, fat tissue, and blood vessels at the external part of the eye cup, the eyes were incubated with 2% Dispase II (neutral protease; Roche Applied Science, Indianapolis, IN) in Hanks’ balanced salt solution (Invitrogen, Waltham, MA) at 37°C for 30 minutes. The explants were kept in the media for 3 days without any treatments until the vessel grew. On the fourth day, explants were treated with PBS (vehicle), 10 mmol/L lactate, or 80 μmol/L 3, 5-dihydroxybenzoic acid until the sixth day. Photographs were taken before the treatment daily until the sixth day. By using ImageJ version 1.53 t, the angiogenesis response was determined by measuring the vessel area and normalizing to the control groups.

### Western Blot Analysis

Collected RPE/choroid complex from aged match tissue samples (PT7, PT9, PT12, and PT15) and from wild-type (WT) and *Hcar1-*KO mice were lysed in radioimmunoprecipitation assay buffer (Cell Signaling Technology, Danvers, MA) supplemented with 0.1 mg/mL phenylmethylsulfonyl fluoride and proteinase inhibitor cocktail (11697498001; Roche), and then were homogenized thoroughly (BRANSON Sonifier 150) and kept at 4°C for 1 hour. After centrifuging at 12,000 × *g* at 4°C for 15 minutes, the supernatant was collected and subjected to protein concentration measurement by Bradford method (Bio-Rad).^27^ Proteins were added to reducing Laemmli sample buffer (Boston BioProducts, Milford, MA) and heated at 95°C, and approximately 40 μg of protein sample was loaded and electrophoresed on SDS-PAGE gel and electroblotted onto polyvinylidene difluoride membranes (Bio-Rad). After blocking with 5% bovine serum albumin (Sigma-Aldrich), the membranes were immunoblotted overnight with primary antibodies (Table 2) at 4°C. After washing, membranes were incubated with their respective secondary antibodies horseradish peroxidase conjugated. Enhanced chemiluminescence (GE Healthcare, Little Chalfont, UK) was detected by using the ImageQuant LAS-500 (GE Healthcare). Image analysis was done with ImageJ version 1.53 t software.

### Growth Factor Microarray

A commercial kit was used to detect the level of various growth factors that are involved in angiogenesis (mouse growth factor array C3; RayBiotech, Peachtree Corners, GA). After blocking the membrane at room temperature, 500 μg of total protein was loaded and incubated overnight at 4°C. After washing the membrane, a biotinylated antibody cocktail was added into each well and incubated overnight at 4°C and detected by horseradish peroxidase–streptavidin and chemiluminescence. Membranes were imaged using the ImageQuant LAS-500. The analysis was done with ImageJ version 1.53 t.

### ROS/RNS Detection

Levels of reactive species were determined with a commercial kit that uses an oxidizable fluorogenic probe, DCFH-DiOxyQ (STA-347-5; Cell Biolabs, San Diego, CA). RPE/choroid complex was homogenized, and then 80 μg of lysate was added to a 96-well plate with a black wall and transparent bottom. The fluorescence was read at 480 nm excitation/530 nm emission. Samples were run in quadruplicate. H_2_O_2_ dilutions were used for the standard curve. The levels of ROS/RNS were calculated and normalized to the amount of protein.

### Superoxide Dismutase Detection

Superoxide dismutase activity was measured by a detection kit that uses the water-soluble tetrazolium salt, WST-1 [2-(4-iodophenyl)- 3-(4-nitrophenyl)-5-(2,4-disulfophenyl)-2Htetrazolium, monosodium salt] (19,160; Sigma-Aldrich). RPE/choroidal complex of a cohort of mice was obtained and homogenized by sonication for 10 seconds in ice-cold buffer, 0.1 mol/L Tris/HCl (pH 7.4) containing 5 mmol/L β-ME, 0.5% Triton X-100, and 0.1 mg/mL phenylmethylsulfonyl fluoride, with a tissue-weight/buffer of the ratio of 1:8 at 4°C. After centrifuging at 14,000 × *g* for 5 minutes at 4°C. Then, supernatants were collected to assay according to the manual. Absorbance was read out at 450 nm. Superoxide dismutase activity was then calculated on the basis of the formula provided by the kit.

### Protein Synthesis Rate Measurement

The nascent protein synthesis rate was measured using Click-&-Go Plus 488 OPP Protein Synthesis Assay Kit (Click-Chemistry Tool, Scottsdale, AZ), according to the manufacturer's protocol. Equal sections of the subretina were dissected and washed in a 6-well plate with media. After 5 hours, O-propargyl-puromycin (OPP) was added, which is an analog of puromycin (can enter the acceptor site of ribosomes and incorporate into nascent polypeptide chains) and then samples were incubated for 30 minutes. The amount of incorporated OPP is identified by a click chemical reaction by AZDye 488 Azide (Alexa Fluor 488 equivalent). Finally, the intensity of AZDye 488 Azide is adjusted with the intensity of DNA counterstain Hoechst 33,342 and indicates the nascent protein synthesis rate.

### Primary RPE Cell Culture

Primary RPE cells were obtained from mice according to a described method^28^ with some modifications. Mice were sacrificed, and their eyes were enucleated. After removing the extra tissues, the obtained eye cups were washed and incubated at room temperature in Dulbecco’s modified Eagle’s medium (Invitrogen) supplemented with 10% fetal bovine serum and 0.2% fungizone (15290026; Thermo Fisher),^29^ to dissociate RPE cells sufficiently from the outer retina, they were kept in the darkness. On the following day, after washing with PBS several times, the eyes were supplemented with a cold mixture containing 2 mg/mL trypsin (T9201; Sigma-Aldrich)/collagenase I (C0130; Sigma-Aldrich) in Dulbecco’s modified Eagle's medium for 30 minutes at 37°C.^30^ After stopping the reaction with Dulbecco’s modified Eagle’s medium containing 10% fetal bovine serum and 1% penicillin/streptomycin, the RPE layer was then separated by peeling from the retina and the choroid by small forceps. The solution of single RPE cells from one eye was gently resuspended and placed in the 24-well plates at a rate of one eye per well that was precoated freshly with an attachment factor (S006100; Gibco, Fisher Scientific, Waltham, MA). Cells were maintained for 1 week before any treatments. The purity of obtained RPE cells was determined by the staining of an RPE marker RPE-65.

### TUNEL Assay

Terminal deoxynucleotidyl transferase-mediated dUTP nick-end labeling (TUNEL) assay was performed by using terminal deoxynucleotidyl transferase fluorescein-dUTP nick end labeling. An assay was applied on eye cryosections based on the kit instructions (Roche). Negative controls were composed of samples without any terminal deoxynucleotidyl transferase from the labeling step, which had no color. Cells including the whole nucleus staining or with circle staining were considered as TUNEL-positive cells, as previously described.^31^

### RPE Flat Mount

Eyes were enucleated and then fixed in 4% paraformaldehyde for 1 hour. Excessive external tissue, blood vessels, cornea, lens, and retina were then delicately removed with forceps under a dissecting microscope. The posterior eye cup (RPE/choroid complex) was incubated with blocking solution (1× PBS, 1% bovine serum albumin, and 0.1% Triton-X) for 1 hour at room temperature, followed by incubation with fluorescein isothiocyanate–phalloidin (1:400; Biotium; San Francisco, CA) at 4°C overnight. On the following day, after washing with PBS, four to five cuts were made to unfold the eyecup and then mounted with a coverslip. Slides were then subjected to confocal microscopy (SP8; Leica).

### Genotypic Screening

Mice were genotyped by using DNA extraction of mice tails. Tails were immersed in a digestion buffer (5 mmol/L EDTA, 200 mmol/L NaCl, 100 mmol/L TRIS-HCl, 0.2% SDS, and 0.4 mg proteinase K) and incubated overnight at 55°C. Samples were then washed and centrifuged by ethanol 100% and 70% at 16,000 × *g* for 30 and 20 minutes, respectively, and finally diluted in TE buffer. DNA was used for genotyping PCR using Taq DNA polymerase. The *Hcar1-*KO mice genotype was confirmed using two different primers. The primer pairs, *Hcar1* WT (forward: 5′-CCATCTCCAACCGGACTGCC-3′; and reverse: 5′-GCTGCAGATTGTGAGCTTGGT-3′) amplified a 512-bp fragment present only in the *Hcar1*^+/+^ mice genome. The other primer, *Hcar1-*Neo (forward: 5′-GCAGCGCATCGCCTTCTATC-3′; and reverse: 5′-GATATCAGGTGGGACAAGTCC-3′), amplified a neomycin sequence plus a section of *Hcar1* gene (808 bp) that exists in *Hcar1-*KO mice.

### Statistical Analysis

Statistical analysis was performed by Prism 9.0 software (GraphPad Software, San Diego, CA). and two-tailed unpaired *t*-test and one-and two-way analysis of variance were used for data analysis. Significance between groups was calculated with Bonferroni *post hoc* analysis. Data were presented as means ± SEM with the statistical significance at ∗*P* < 0.05, ∗∗*P* < 0.01, ∗∗∗*P* < 0.001, and ∗∗∗∗*P* < 0.0001.

## Results

### HCAR1 Signaling in RPE Cells Promotes Angiogenesis in the Developing Choroid

**Figure 1:**
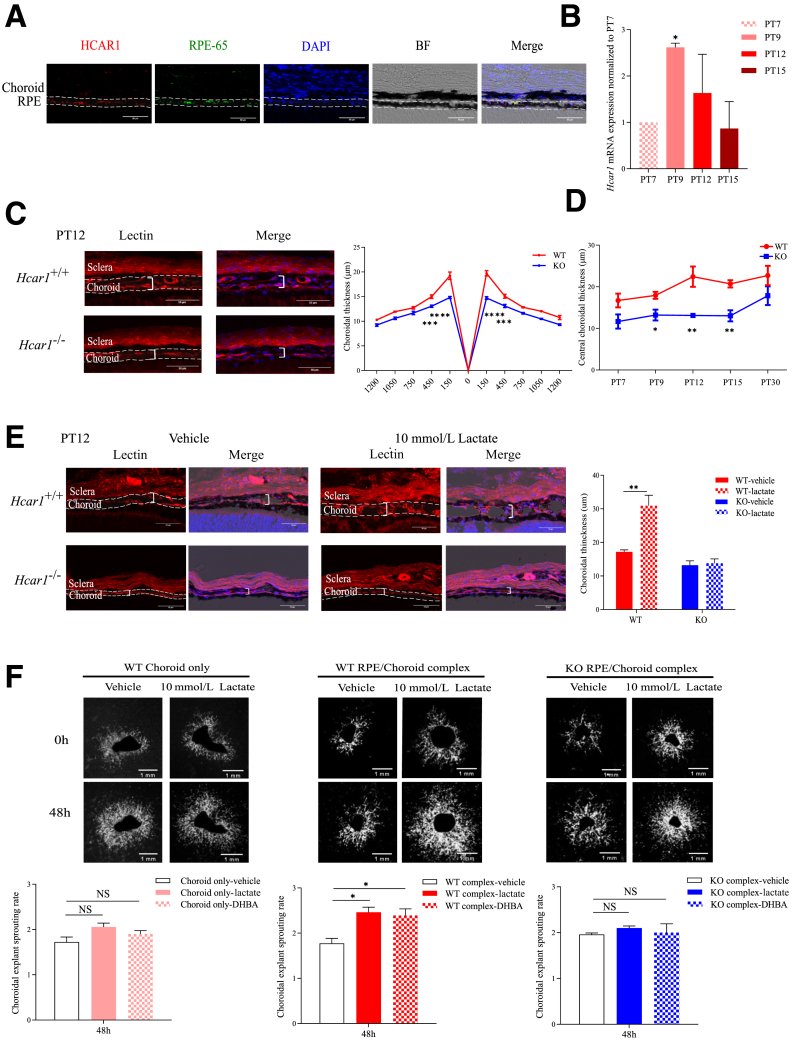
HCAR1 expression in retinal pigment epithelium (RPE) cells promotes choroidal angiogenesis. **A:** Representative confocal images of subretinas from PT12 pups, stained with anti-HCAR1 (red) and anti-RPE65 (green); nuclei were counterstained with DAPI (blue). HCAR1 and RPE signals exhibit strong colocalization. The RPE cell layer is delineated with a **dashed line**. **B:** mRNA levels of *Hcar1* in the RPE/choroid layers were measured at PT7, PT9, PT12, and PT15 by quantitative RT-PCR. **C: Left panels:** Representative confocal microscopy images of choroidal cross-sections from PT12 pups, stained with rhodamine-conjugated *Griffonia simplicifolia* lectin I (red). Choroidal thickness is delineated with a **dashed line**; examples of measured thicknesses are shown with plain white brackets. **Right panels:** Spider graphs reveal quantification of the choroid thickness at indicated distance (μm) from the optic nerve (position 0) in PT12 pups. The values of choroid thickness are provided in μm. **D:** Quantifications of central choroidal thickness at PT7, PT9, PT12, PT15, and PT30 in wild-type (WT) and *Hcar1*-knockout (KO) mice. **E: Left panels:** Representative images of subretinas stained with rhodamine-conjugated *Griffonia simplicifolia* lectin I (red) from PT12 mice injected intravitreally with 10 mmol/L lactate at PT9. **Right panel:** Quantification of the choroidal vasculature thickness on lactate injection. Choroidal thickness is delineated with a **dashed line**; examples of measured thicknesses are shown with plain white brackets. **F: Top panels:** Representative images of *ex vivo* choroid sprout assays using isolated choroid or RPE/choroid punches from WT mice, and RPE/choroid from KO mice treated with vehicle or 10 mmol/L lactate for 48 hours. **Bottom panels:** Quantification of the choroid sprout assays treated with vehicle, 10 mmol/L lactate, or 80 μmol/L 3, 5-dihydroxybenzoic acid (DHBA) for 48 hours. The explant sprouting area at 48 hours was normalized to the choroidal explant area at 0 hours. Data are presented as means ± SEM (**B**–**F**). *n* = 4 to 6 per group. ∗*P* < 0.05, ∗∗*P* < 0.01, ∗∗∗*P* < 0.001, and ∗∗∗∗*P* < 0.0001. Scale bars: 50 μm (**A**, **C**, and **E**); 1 mm (**F**). BF, bright field; NS, not significant.

Immunohistofluorescence revealed localization of HCAR1 specifically in RPE (colocalized with RPE65) in young pups (Figure 1A). These results were corroborated by *Hcar1* mRNA expression specifically on primary RPE, but not on choroid of WT mice (Supplemental Figure S1, A–C). mRNA expression of *Hcar1* peaked at PT9 (Figure 1B) in young pups. *Hcar1*^*−/−*^ mice displayed thinner choroid at PT12 compared with their WT littermates, specifically in the central region (Figure 1C). Murine choroidal thickness grows steadily post-natally to peak at 8 weeks after birth.^2^ In contrast to WT mice, *Hcar1*^*−/−*^ animals did not display any increase in critically important central choroidal thickness over the course of the first 2 weeks of life (Figure 1D). This resulted in a thinner central choroid leading to substantially decreased O_2_ and nutrient delivery to the outer retina in *Hcar1*^*−/−*^ mice, as observed with ischemia associated with choroidal thinning. This central choroid vulnerability results from amplified oxidative stress in the neonate.^32^ After PT15, the choroidal thickness in *Hcar1*^*−/−*^ subjects catches up to normal thickness.^33^ Growth of the peripheral choroid did not differ between WT and *Hcar1*^*−/−*^ mice. Of relevance, vortex vein size appeared comparable in WT and *Hcar1*^*−/−*^ animals (Supplemental Figure S1D), thus unlikely to contribute to differences in choroidal thickness. Conversely, stimulation of the choroid of WT mice with lactate (intravitreal at PT9) triggered a robust thickening of the choroid (Figure 1E), which, as expected, was not observed in mice deficient in HCAR1. Although the RPE harbors HCAR1, its morphology is unaltered in *Hcar1*^*−/−*^, as witnessed on phalloidin-stained flat mounts (Supplemental Figure S1E). On the other hand, the RPE is essential in governing the angiogenic response of the choroid to lactate stimulation. This inference was confirmed on subretinal explants, which revealed lactate- and 3, 5-dihydroxybenzoic acid– (specific HCAR1 agonist)^34^ induced sprouting only in explants containing both RPE and choroid, but not choroid on its own (Figure 1F). RPE-choroid complex of HCAR1-null mice was unresponsive to lactate. Taken together, *in vivo* and *ex vivo* results suggest that lactate sensing by the HCAR1 receptor, residing in the RPE cells, promotes angiogenesis of immediately adjacent choroid.

### Mechanisms Associated with Defective Choroidal Growth in HCAR1-Null Subjects

Given that inflammation can be detrimental to the subretina^35^^,^^36^ and HCAR1 is reported to mitigate innate immune response,^19^^,^^20^ inflammatory cell invasion of the central subretina was assessed. Few ionized calcium-binding adaptor molecule 1 (Iba1)^+^ mononuclear phagocytes were detected in both WT and HCAR1-null mice during the time when the choroid failed to grow (Supplemental Figure S2A), including the central choroidal regions most affected by *Hcar1*-KO (Supplemental Figure S2A). In addition, other than an increase in mRNA of proinflammatory IL-1β along with that of anti-inflammatory IL-10 at PT9, other inflammatory factors were hardly changed in HCAR1-null compared with WT mice. At PT12, there tended to be a suppression of inflammatory markers in HCAR1-null animals (Supplemental Figure S2B). Together, these observations do not support a role for inflammation in central choroidal thinning in HCAR1-null mice.

Cell death was assessed in the pursuit of mechanisms potentially implicated in choroidal thinning in HCAR1-null mice. TUNEL positivity was scarcely detected in the central subretina (Supplemental Figure S3A). Similarly, local expression of the apoptotic/necroptotic factors B-cell lymphoma 2 (BCL2), BCL2-associated agonist of cell death (BAD), caspase 3 (CASP3), Fas ligand (FASL)*,* and receptor-interacting protein kinase 3 (RIPK3) did not differ between HCAR1-null and WT animals (Supplemental Figure S3B).

**Figure 2:**
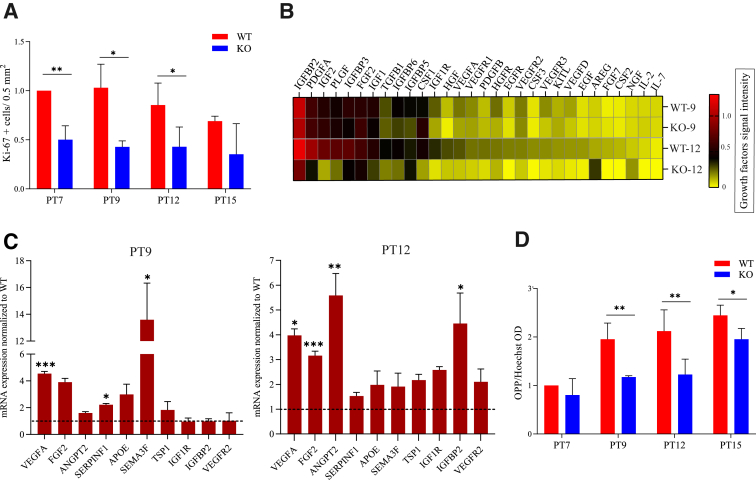
HCAR1 deficiency impairs cell proliferation and protein synthesis. **A:** Quantification of number of Ki-67–positive cells per 0.5 mm^2^ in central choroid at PT7, PT9, PT12, and PT15 pups; representative images are shown in Supplemental Figure 4A. **B:** Heat map of expression levels of several growth factors involved in cellular proliferation in wild-type (WT) and *Hcar1*-knockout (KO) mice at PT9 and PT12. Protein levels were analyzed by protein microarray, and the signal intensity of each growth factor was normalized to the internal positive control; data were not subsequently transformed. **C:** mRNA levels of 10 angiogenic factors were measured by quantitative RT-PCR; data presented as fold changes of the KO relative to the WT pups, at PT9 (**left panel**) and PT12 (**right panel**). (The **dotted line** indicates the average gene expression in WT that was considered as 1.) **D:** O-propargyl-puromycin (OPP) incorporation, as a proxy of protein translation rate, was revealed by the AZDye 488 Azide. The AZDye 488 signal was quantified on confocal microscopy images and adjusted relatively to the intensity of the Hoechst DNA counterstain; representative images are shown in Supplemental Figure 4C. Data are presented as means ± SEM (**A**, **C**, and **D**). *n* = 3 to 6 per group. ∗*P* < 0.05, ∗∗*P* < 0.01, and ∗∗∗*P* < 0.001. ANGPT2, angiopoietin 2; APOE, apolipoprotein E; AREG, amphiregulin; CSF, colony-stimulating factor; EGF, epidermal growth factor; EGFR, epidermal growth factor receptor; FGF, fibroblast growth factor; HGF, hepatocyte growth factor; HGFR, hepatocyte growth factor receptor; IGF, insulin-like growth factor; IGF1R, insulin-like growth factor 1 receptor; IGFBP, insulin-like growth factor binding protein; KITL, KIT ligand; NGF, nerve growth factor; PDGF, platelet-derived growth factor; PLGF, placental growth factor; SEMA3F, semaphorin 3F; SERPINF1, serpin family F member 1; TGFB1, transforming growth factor beta 1; TSP1, thrombospondin 1; VEGF, vascular endothelial growth factor; VEGFR, vascular endothelial growth factor receptor.

Because cell loss could not explain a thinner choroid in HCAR1-null mice, a reduced choroidal proliferation rate was determined in the vasculature. Concordantly, expression of the proliferation marker Ki-67 on ocular cross-sections was lower in HCAR1*-*null mice compared with WT mice (Figure 2A and Supplemental Figure S4A). Consistently, given the critical role of RPE in eliciting choroidal vascular sprouting (Figure 1F), growth factor protein expression was lower in primary RPE cells from HCAR1-null mice (Supplemental Figure S4B). Protein microarray analysis also revealed a decrease in growth factor expression in central subretina of HCAR1-null compared with WT mice, to some extent at PT9 and particularly at PT12 (Figure 2B). mRNA expression of several growth factors, such as VEGFA, serpin family F member 1 (SERPINF1), semaphorin 3F (SEMA3F), FGF2, angiopoietin 2 (ANGPT2), and insulin-like growth factor binding protein 2 (IGFBP2) was increased at PT9 and PT12 in HCAR1-null versus WT mice (Figure 2C). Whether depressed growth factor proteins reflected a global protein translation defect was studied next. *Ex vivo*, subretinal protein translation rate determined using OPP incorporation into proteins^37^ was substantially lower in HCAR1-null versus that in WT mice (Figure 2D and Supplemental Figure S4C).

**Figure 3:**
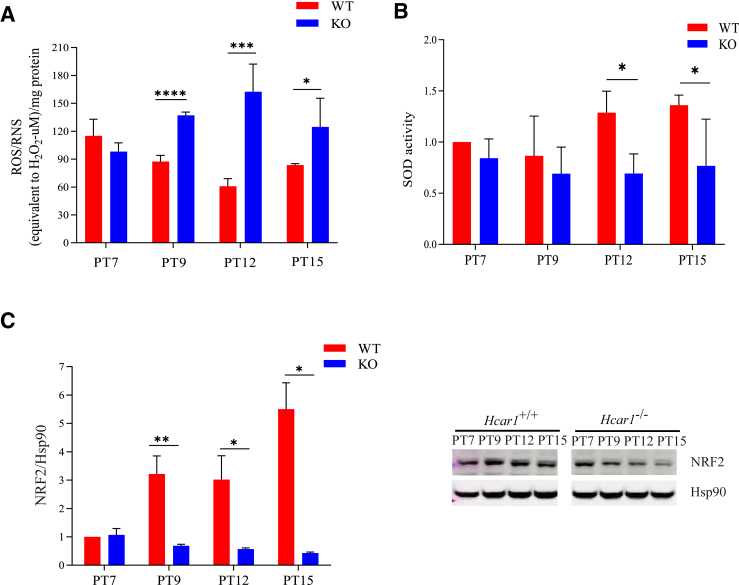
HCAR1 deficiency leads to a higher level of oxidative stress in the outer retina. **A:** Reactive oxygen species (ROS) and reactive nitrogen species (RNS) were measured in freshly isolated retinal pigment epithelium (RPE)/choroid from wild-type (WT) and *Hcar1*-knockout (KO) pups at PT7, PT9, PT12, and PT15. ROS and RNS levels were adjusted to the amount of tissue collected. **B:** Superoxide dismutase (SOD) activity in freshly isolated RPE/choroid from WT and KO pups at PT7, PT9, PT12, and PT15. **C: Left panel:** Quantification. **Right panel:** Representative Western blot analysis images of the stress sensor nuclear factor erythroid 2-related factor 2 (NRF2) in the RPE/choroid from WT and KO pups at PT7, PT9, PT12, and PT15, relative to heat shock protein 90 (Hsp90) expression. Data are presented as means ± SEM (**A**–**C**). *n* = 4 to 6 per group (**A**–**C**). ∗*P* < 0.05, ∗∗*P* < 0.01, ∗∗∗*P* < 0.001, and ∗∗∗∗*P* < 0.0001.

Protein translation rate can be affected by various cellular stress pathways,^38^ notably oxidative stress.^39^^,^^40^ Assessment of reactive oxygen and nitrogen species (ROS, RNS) in subretina of mouse pups revealed higher levels in *Hcar1*^*−/−*^ mice than in WT mice, along with the lower activity of major antioxidant, superoxide dismutase, and of major transcription factor regulator of oxidation-reduction homeostasis, nuclear factor erythroid 2-related factor 2 (NRF2) (Figure 3). Together, these results corroborate HCAR1's role in regulating the oxidation-reduction state,^41^ as observed here in the RPE/choroid complex during choroidal development.

### HCAR1 Deficiency and Endoplasmic Reticulum Stress in the RPE/Choroid Complex

**Figure 4:**
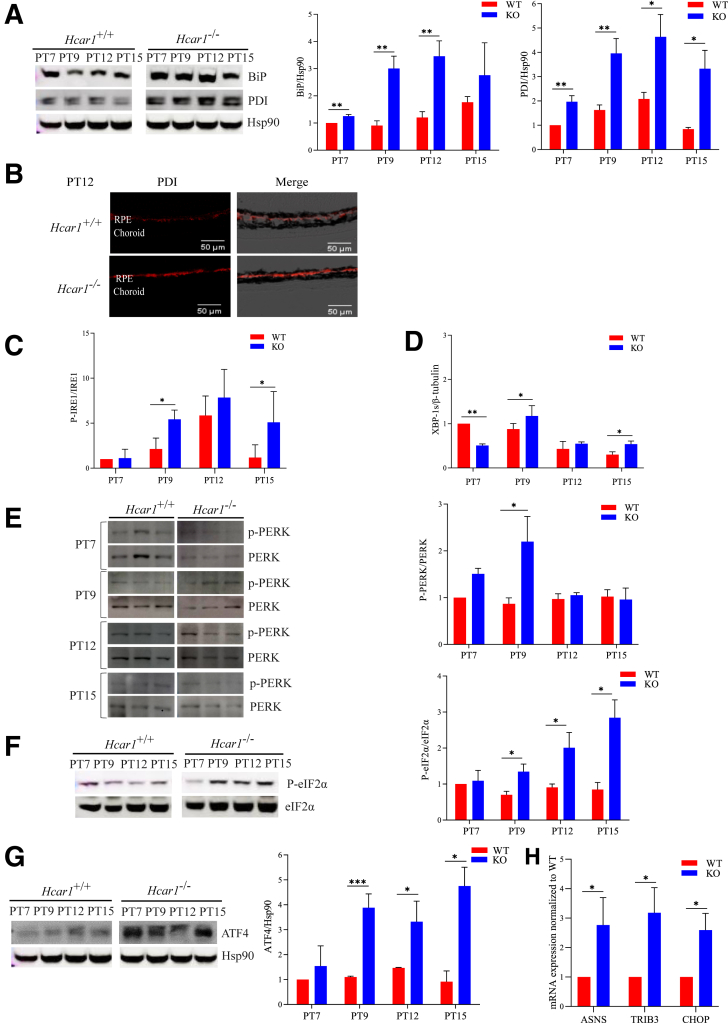
HCAR1 maintains endoplasmic reticulum (ER) homeostasis in the retinal pigment epithelium (RPE)/choroid complex. **A:** Representative Western blot analysis images of the ER stress markers binding immunoglobulin protein (BiP) and protein disulfide isomerase (PDI), from the subretina of wild-type (WT) and *Hcar1*-knockout (KO) mice at PT7, PT9, PT12, and PT15. Immediately to the right is the Western blot analysis quantification for BiP and PDI. **B:** Representative confocal images of subretinas from PT12 pups, stained with anti-PDI (red). **C:** Quantification of the phosphorylated IRE-1α (P-IRE-1α) relative to the total IRE-1α signal, in the subretina of WT and *Hcar1*^*−/−*^ mice at PT7, PT9, PT12, and PT15. Representative images of IRE-1α are in Supplemental Figure 5B. **D:** Quantification of the level of spliced XBP1 (XBP-1s) relative to the level of β-tubulin, in the subretina of WT and *Hcar1*^*−/−*^ mice at PT7, PT9, PT12, and PT15; for representative image of XBP-1s, see Supplemental Figure 5C. **E:** Representative Western blot analyses of phosphorylated ER stress signal protein kinase RNA-like ER kinase (PERK), from the subretina of WT and *Hcar1*^*−/−*^ mice at PT7, PT9, PT12, and PT15. Immediately adjacent (**right**) is the quantification of phosphorylated PERK (P-PERK) relative to total PERK. **F:** Representative Western blot analysis of phosphorylated eukaryotic translation initiation factor 2α (P-eIF2α) relative to total eIF2α in subretina of WT and *Hcar1*^*−/−*^ mice at PT7, PT9, PT12, and PT15. Histogram (**right**) is the quantification of eIF2α phosphorylation Western blot analysis, presented as the ratio of the phosphorylated eIF2α signal/the total eIF2α signal. **G:** Representative Western blot analysis and quantifications (histogram) of activating transcription factor 4 (ATF4), from the subretina of WT and *Hcar1*-KO (KO) mice at PT7, PT9, PT12, and PT15. **H:** Quantitative RT-PCR quantifications of ATF4 downstream target gene expression in the subretina of *Hcar1*^*−/−*^ and WT mice at PT12. Data are presented as means ± SEM (**A** and **C**–**H**). *n* = 4 (**A**, **B**, **F**, **G**, and **H**) to 6 (**C**–**E**) per group. ∗*P* < 0.05, ∗∗*P* < 0.01, and ∗∗∗*P* < 0.001. Scale bars = 50 μm (**B**). ASNS, asparagine synthetase; CHOP, C/EBP homologous protein; Hsp90, heat shock protein 90; TRIB3, tribbles homolog 3.

Cell dysregulation impacting proliferation was investigated because of the co-occurrence of oxidative stress with choroidal thinning during the second week of post-natal life. Discrepant translation and transcription^42^^,^^43^ in the phase of oxidant stress motivated consideration of endoplasmic reticulum (ER) stress (Supplemental Figure S5A).^38^^,^^39^^,^^44^ Major ER stress markers binding immunoglobulin protein [binding immunoglobulin protein (BiP)/glucose regulated protein 78 (GRP78)] and protein disulfide isomerase notably at the RPE (Figure 4, A and B) were clearly increased, along with activation of cytoprotective-attempted unfolded protein response sensors (inositol-requiring enzyme-1 and downstream X-box binding protein-1) (Figure 4, C and D, and Supplemental Figure S5, B and C). The major ER stress effector protein kinase RNA-like ER kinase (PERK) was also rapidly activated (Figure 4E), which led to phosphorylation and, in turn, inhibition of the global translation eukaryotic translation initiation factor 2α (eIF2α) (Figure 4F).^45^^,^^46^ This resulted in activation of activating transcription factor (ATF) 4 (Figure 4G) substantiated by increased downstream expression of growth-promoting cap-independent *Asns*, *Trib3*, and *Chop* (Figure 4H), in an (insufficient) attempt to compensate for global cap-dependent deficient translation. Expression of the other unfolded protein response sensor, activating transcription factor 6 (ATF6), was hardly altered in *Hcar1*^−/−^ mice (Supplemental Figure S5D). Taken together, these data indicate that *Hcar1*-KO leads to an increased ER stress and activation of the downstream unfolded protein response response in the subretina.

### ISR Inhibitor Reverses Translation Inhibition and Choroidal Thinning in *Hcar1*^*−/−*^ Mice

The integrated stress response (ISR) is a complex adaptive signaling pathway that responds to diverse stresses, including extrinsic (eg, hypoxia) and intrinsic factors (such as oxidative and ensued ER stress).^43^ eIF2α is the core factor in the ISR, allowing cells to adapt to environmental and pathologic stimuli.^43^ In addition to causing cap-dependent global inhibition of translation, eIF2α phosphorylation^47^ induces translation of cap-independent specific downstream effectors of the ISR, among which the transcription factor activating transcription factor 4 (*Atf4*) is the best characterized.^48^ Western blot analyses of ATF4 levels in the RPE/choroid of *Hcar1*^*−/−*^ KO pups revealed increased ATF4 expression compared with age-matched WT counterparts (Figure 4G). In addition, target genes downstream of ATF4, including prosurvival asparagine synthetase (*Asns*), tribbles homolog 3 (*Trib3*), and C/EBP homologous protein (*Chop/Ddit3*), were coincidentally increased at PT12 ^49^^,^^50^ (Figure 4H), consistent with increased activation of the ISR.

**Figure 5:**
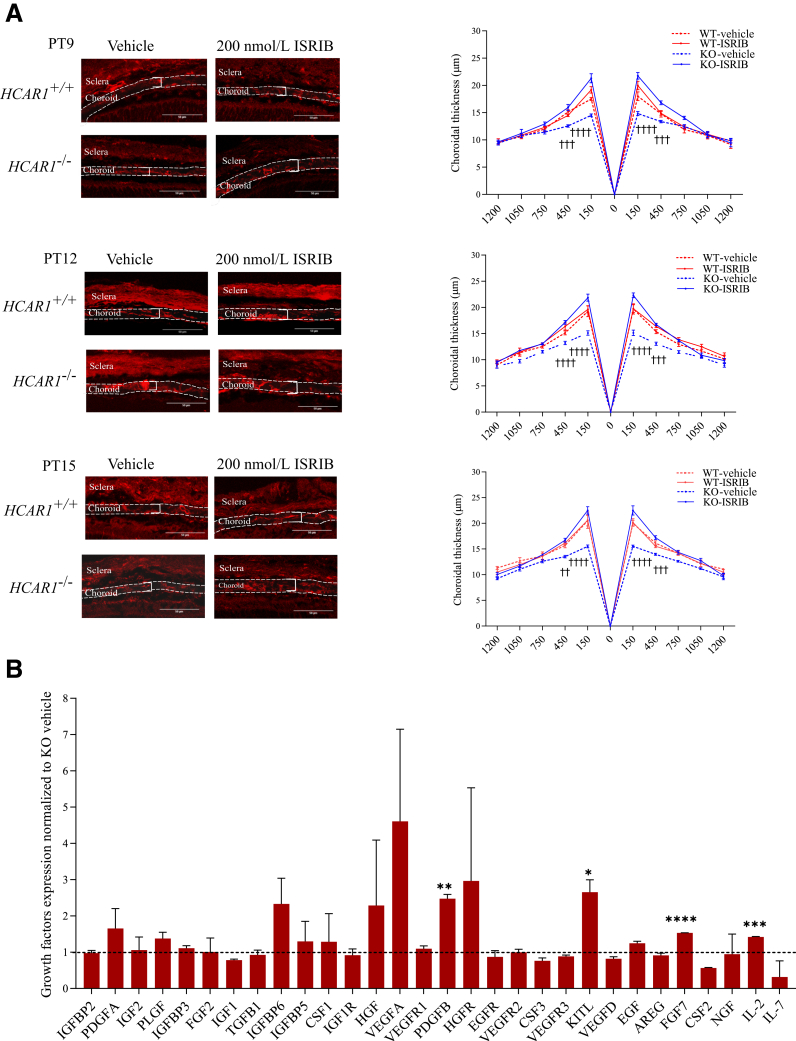
Integrated stress response inhibitor (ISRIB) rescues the choroidal involution in *Hcar1-*knockout (KO) mice. **A:** Representative confocal images of a rhodamine-conjugated *Griffonia simplicifolia* lectin I staining of choroidal sections from PT9, PT12, or PT15 mice intravitreally injected with ISRIB (200 nmol/L) or vehicle at PT7, PT9, and PT12. Choroidal region is delineated with a **dashed line**; examples of measured thicknesses are shown with plain white brackets. To the immediate right are spider graph quantifications of the choroid thickness at indicated distance (μm) from the optic nerve (position 0) in the four groups [wild type (WT)-ISRIB, WT-vehicle, KO-ISRIB, and KO-vehicle] at ages indicated (PT9, PT12, and PT15). **B:** Growth factor expression at PT12 on injection of ISRIB at PT9 in KO mice, relative to similarly treated WT animals. (The **dotted line** indicates the average growth factor expression in KO vehicle that was considered as 1.) Data are presented as means ± SEM (**B**). *n* = 3 to 6 per group ∗*P* < 0.05, ∗∗*P* < 0.01, ∗∗∗*P* < 0.001, and ∗∗∗∗*P* < 0.0001; ^††^*P* < 0.01, ^†††^*P* < 0.001, and ^††††^*P* < 0.0001 compared with KO-ISRIB and KO-vehicle groups. Scale bars = 50 μm (**A**). AREG, amphiregulin; CSF, colony-stimulating factor; EGF, epidermal growth factor; EGFR, epidermal growth factor receptor; FGF, fibroblast growth factor; HGF, hepatocyte growth factor; HGFR, hepatocyte growth factor receptor; IGF, insulin-like growth factor; IGF1R, insulin-like growth factor 1 receptor; IGFBP, insulin-like growth factor binding protein; KITL, KIT ligand; NGF, nerve growth factor; PDGF, platelet-derived growth factor; PLGF, placental growth factor; TGFB1, transforming growth factor beta 1; VEGF, vascular endothelial growth factor; VEGFR, vascular endothelial growth factor receptor.

Inhibition of the ISR, which should restore protein translation and ensuing cell proliferation, would rescue choroidal thinning in the *Hcar1*^*−/−*^ mice. For this purpose, the ISR inhibitor (ISRIB) was used—a small molecule that renders the assembly of the pre-initiation complex insensitive to the phosphorylated eIF2α, thus enabling translation.^51^^,^^52^ ISRIB was injected intravitreally into PT7, PT9, and PT12 mice, and choroidal thickness was evaluated at PT9, PT12, and PT15. Although, as expected, WT mice were unresponsive to ISRIB, the latter led to a significant increase in the choroidal thickness in *Hcar1*-KO mice at all ages studied (Figure 5A). Inhibition of the ISR pathway at PT12 in *Hcar1*^*−/−*^ mice resulted in a rescue of the protein expression of several growth factors lost on *Hcar1-*KO, such as platelet-derived growth factor B, KIT ligand (KITL), FGF7, and IL-2, and normalized expression of many others (Figure 5B), compared with suppressed growth factors in untreated animals (Figure 2B). As expected, WT mice were unresponsive to ISR inhibition. Together, these observations confirmed that eIF2α phosphorylation associated with ISR restricts normal choroidal development in the phase of depleted *Hcar1.*

## Discussion

Integrity of the choroid is essential for vision; conversely, choroidal involution has dire consequences on vision acuity as is the case in aging with macular degeneration, diabetic retinopathy, and in young subjects with retinopathy of prematurity.^1^^,^^33^^,^^53^^,^^54^ The choroid is primarily a vascular tissue estimated to contain the highest blood flow in the body, essential to supply O_2_ and nutrients to the highly metabolic outer retina, particularly as it applies to the macula. Accordingly, maintenance of choroidal vasculature requires a healthy RPE, which is a major source of angiogenic factors (such as VEGFA, FGF2, TGFB1, IGF-1, and platelet-derived growth factor),^4^^,^^55^^,^^56^ necessary for the choroidal integrity. RPE cells are major metabolic generators of lactate. Along with a variety of other carbohydrate, protein, and lipid metabolites, lactate is not simply a metabolic end product but a ligand for a specific G-protein–coupled receptor (namely, HCAR1). HCAR1 plays an important role in vascular development of the retina and brain.^15^^,^^21^ On the basis of the proximity and interaction of the choroid with the lactate-rich RPE, a role for HCAR1 on choroidal integrity during development was surmised. Herein, HCAR1 played a role beyond that of its ligand as a mere metabolic product—importantly as a major contributor to the development of the choroid.

HCAR1 is expressed on RPE cells (but not on choroid), yet it is essential for proper choroidal development and angiogenesis. Accordingly, stimulation of HCAR1 elicits choroidal vascular sprouting only when RPE is present adjacent to the choroid, inferring paracrine release of angiogenic factors from the RPE. Conversely, the absence of HCAR1 curtails growth factor release and in turn compromises developmental choroidal growth. The expression of HCAR1 in the RPE layer makes it an ideal location to sense the metabolic needs of the outer retina, including during development and on different light exposures.^57^ This metabolic surveillance mediated by the lactate receptor coordinates choroid development to permit adequate nutrient delivery to the retina; on the basis of Poiseuille's law, the approximately 35% decrease in choroidal thickness observed in HCAR1-deficient mice corresponds to a marked, approximately 80%, decrease in local hemodynamics. Hence, when HCAR1 is absent, lactate sensing is impaired, leading to a decreased translation rate, thus lowering the expression of several growth factors, in turn impairing choroidal thickening. The absence of HCAR1 expression leads to curtailed inner retina vascularization, defective transduction of photoreceptor signaling, as assessed by electroretinogram, and defective retinal ganglion cell projections toward the central nervous system.^15^^,^^58^ Although a mechanism originating from the retina in contributing to choroidal involution in *Hcar1*^*−/−*^ mice cannot be fully excluded, a major role for the RPE on choroidal growth (sprouting) is substantiated *ex vivo*. The present study extends the concept that metabolites of different metabolic pathways exert cellular actions by acting as ligands of specific receptors, as is the case for oxidative pathway intermediates [eg, HCAR1, succinate receptor 1 (Sucnr1), and G-protein-coupled receptor 99 (GPR99)], lipid metabolism [eg, free fatty acid receptor 1 (FFAR1), FFA2, FFA3, and FFAR4], amino acids [eg, calcium-sensing receptor (CaSR)], and others.^59^ HCAR1 belongs to this cluster of G-protein–coupled receptors. In this context, one could presume that metabolic effects incurred by HCAR1 in other organs (eg, adipose tissue) affect integrity of the choroid. A similar reasoning has been made in O_2_-induced retinopathy wherein generalized growth deficiency contributes to aggravation of the retinal vasoobliteration.^60^ However, the body weight of *Hcar1*-KO was comparable to that of WT mice.^16^

A feature of HCAR1 deficiency downstream effects applies to curbed protein translation and ensued diminished growth factor release associated with ER stress, which is somewhat related to oxidant stress. HCAR1 signals via extracellular signal-regulated kinase 1/2 and Akt,^59^ which are important in growth and cytoprotection^61^^,^^62^ by affecting cellular energy in part via ribosome biogenesis.^63^ The latter also plays a critical role in appropriate protein folding. Accordingly, defective HCAR1 expression and signaling elicit unfolded protein response. Interestingly, immunohistochemical OPP incorporation reveals that the impaired protein translation deficiency seems distributed to the RPE/choroid complex and not only the RPE, suggesting that the ER stress conveyed by the RPE impacts the growth factor–deficient choroid in HCAR1-null animals through paracrine manner. By inhibiting ER stress-triggered phosphorylated-eIF2α, critical for global protein translation, one is able to rescue developmental choroidal vascular thickness. Although such rescue was coherently associated with the restoration of protein translation of various growth factors, KITL and platelet-derived growth factor B were notably increased; these factors exert major roles in sustaining the integrity of the vasculature.^64^, ^65^, ^66^, ^67^

The present study uncovers an unprecedented role for HCAR1 in choroidal vascular development, such that in its absence ER stress and ensuing defective protein translation interfere with the growth of this important vascular tissue. Restoration of protein translation preserves the choroid and as a consequence outer retina integrity (Supplemental Figure S6).

## Disclosure Statement

S.C. holds a Canada Research Chair (in vision research) and Leopoldine Wolfe Chair in Translational Research in Vision.

## Supplemental Data

Supplemental Figure S1 *Hcar1**-*knockout (KO) does not affect the retinal pigment epithelium (RPE) morphology. **A:** RT-PCR showing expression of *Hcar1* in the isolated RPE/choroid complex from wild-type (WT) mice, but not in *Hcar1*-KO (KO) mice. **B:** RT-PCR of *Hcar1* mRNA in the isolated choroid and isolated primary RPE (PRPE) cells; HCAR1 is exclusively expressed in the RPE layer. **C:** Confocal images of isolated PRPE staining with RPE65 (red); the nuclei of cells were counterstained with DAPI (blue). **D:** Visualization of vortex veins in the choroid from WT and *Hcar1*-KO (KO) pups at PT12 using podocalyxin (red) and α-smooth muscle actin (α-SMA; green). Four vortex veins are spotted at the equator of the choroid (**white arrows**). **E: Top panels:** Representative confocal images of the RPE layer from WT and *Hcar1*-KO (KO) mice at PT7, PT9, PT12, and PT15 stained with fluorescein isothiocyanate–phalloidin. **Bottom panel****:** Histogram of average RPE cell area measured in the center of the eye cup. Data are presented as means ± SEM (**E**). *n* = 3 (**A**–**C**) to 4 (**D**) per group. Scale bars: 20 μm (**C**); 1 mm (**D**); 50 μm (**E**).

Supplemental Figure S2 Markers of inflammation in subretina of *Hcar1*-knockout (KO) and wild-type (WT) mice during development. **A: Top panels:**. Representative Iba-1 immunohistofluorescence (red) confocal images on cross-sections from WT and *Hcar1*-KO (KO) pups at PT7, PT9, PT12, and PT15; nuclei are counterstained with DAPI (blue). Subretina is delimited by a **dashed line**. **Bottom panel****s****:** Quantification of Iba-1–positive cells in the subretina relative to the observed area, for each corresponding genotype and age and normalized to PT7 WT (**left panel**). Quantification of Iba-1–positive cells in the subretina relative to the central area, for each corresponding genotype and age and normalized to PT7 WT (**right panel**). **B:** mRNA levels of inflammatory factors measured by quantitative RT-PCR and presented as fold change in KO relative to WT animals, at PT9 (**left panel**) and PT12 (**right panel**). (The **dotted line** indicates the average gene expression in WT that was considered as 1.) Data are presented as means ± SEM (**A** and **B**). *n* = 3 to 6 per group. ∗∗*P* < 0.01, ∗∗∗*P* < 0.001. Scale bars = 50 μm (**A**). ONL, outer nuclear layer; RPE, retinal pigment epithelium.

Supplemental Figure S3 Absence of an increase in apoptosis in subretina of young *Hcar1**-*knockout (KO) mice. **A: Top panels:** Terminal deoxynucleotidyl transferase-mediated dUTP nick-end labeling (TUNEL) staining of subretina. Representative confocal images on cross-sections from wild-type (WT) and *Hcar**1*-KO (KO) pups at PT7, PT9, PT12, and PT15; TUNEL-positive cells are shown in green and are highlighted with **white arrows**, and DAPI-stained nuclei are shown in blue. Subretina is delineated by a **dashed line**. **Bottom panel:** Quantification of TUNEL-positive cells in the subretina relative to the observed surface area, for each corresponding genotype and age and normalized to WT at PT7. **B:** mRNA levels of apoptotic factors measured by quantitative RT-PCR and presented as fold change in KO relative to WT animals, at PT9 (**left panel**) and PT12 (**right panel**). (The **dotted line** indicates the average gene expression in WT that was considered as 1.) Data are presented as means ± SEM (**A** and **B**). *n* = 3 to 6 per group. Scale bars = 50 μm (**A**). ONL, outer nuclear layer; RPE, retinal pigment epithelium.

Supplemental Figure S4 *Hcar1*-knockout (KO) mice display a reduction in proliferation rate and protein synthesis. **A:** Representative confocal images of immunohistofluorescence of Ki-67 (green) on ocular cross-sections from wild-type (WT) and *Hcar1*-KO (KO) pups at PT7, PT9, PT12, and PT15; DAPI-stained nuclei are in blue. Subretina is delineated by a **dashed line**. **White arrows** point to single Ki-67–positive cells. **B:** Expression of growth factors was analyzed in primary retinal pigment epithelium (RPE) cells by Western blot analysis. Signal intensity from primary retinal pigment epithelium (pRPE) cells of KO mice was normalized to signal from pRPE of WT animals. (The **dotted line** indicates the average growth factors expression in WT that was considered as 1.) **C:** Representative confocal images of OPP-AZDye 488 incorporation (green); DNA was counterstained with Hoechst (blue). Data are presented as means ± SEM (**B**). *n* = 3 to 6 per group. ∗*P* < 0.05, ∗∗*P* < 0.01, and ∗∗∗*P* < 0.001. Scale bars = 50 μm (**A** and **C**). ONL, outer nuclear layer; Sc., sclera.

Supplemental Figure S5 Activation of unfolded protein response pathways in *Hcar1*-knockout (KO) mice. **A:** Schematic overview of the unfolded protein response pathway. Generated using BioRender.com (Toronto, ON, Canada). **B:** Representative Western blot analyses of phosphorylated inositol-requiring enzyme 1α (P-IRE-1α) compared with total IRE-1α in the subretina of wild-type (WT) and *Hcar1*^*−/−*^ mice at PT7, PT9, PT12, and PT15. **C:** Representative Western blot analyses of spliced X-box binding protein-1 (XBP-1s) in the subretina of WT and *Hcar1*^*−/−*^ mice at PT7, PT9, PT12, and PT15. **D: Left panel:** Representative Western blot analysis of cleaved activating transcription factor 6 (c-ATF6) in the subretina of WT and *Hcar1*^*−/−*^ mice at PT7, PT9, PT12, and PT15. **Right panel:** Quantification of cleaved ATF-6 (c-ATF6) relative to unprocessed ATF6, in the subretina of WT and *Hcar1*^*−/−*^ mice at PT7, PT9, PT12, and PT15. BiP, binding immunoglobulin protein; eIF2α, eukaryotic translation initiation factor 2α; ER, endoplasmic reticulum; PDI, protein disulfide isomerase; PERK, protein kinase RNA-like ER kinase; ROS, reactive oxygen species.

Supplemental Figure S6 Suggested model of the pathways affected by a lack of the *Hcar1* gene. Proposed model depicting the pathways affected by the knockout of *Hcar1* gene. HCAR1 deficiency leads to endoplasmic reticulum (ER) and oxidative stress, resulting in eukaryotic translation initiation factor 2α (eIF2α) phosphorylation and subsequent integrated stress response (ISR) pathway activation. ISR activation and consequent inhibition of protein translation leads to a decrease in growth factors expression, which, in turn, results in choroidal thinning. ISR inhibitor (ISRIB) rescues growth factor expression and choroidal thickness. Image generated using BioRender.com (Toronto, ON, Canada). RPE, retinal pigment epithelium.
