# Supplementary figures and images for: Endoplasmic Reticulum Stress Delays Choroid Development in the HCAR1 Knockout Mouse
Source: Am J Pathol. 2024 Sep 26;194(12):2382–97. doi: 10.1016/j.ajpath.2024.09.002 (PMC12179509; doi:10.1016/j.ajpath.2024.09.002)

**Fig S1**

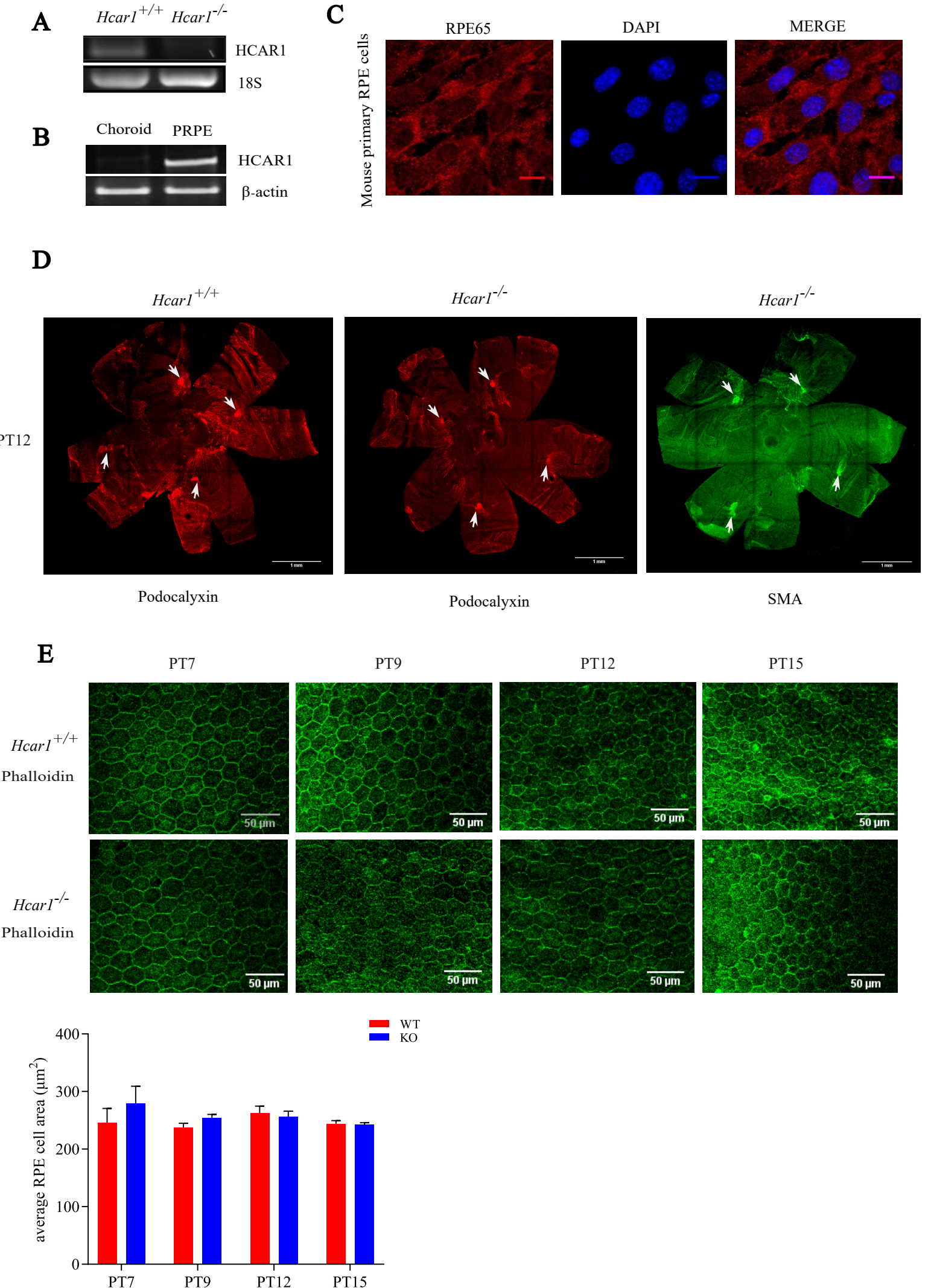

Supplement: Supplemental Figure S1 — Hcar1-knockout (KO) does not affect the retinal pigment epithelium (RPE) morphology. A: RT-PCR showing expression of Hcar1 in the isolated RPE/choroid complex from wild-type (WT) mice, but not in Hcar1-KO (KO) mice. B: RT-PCR of Hcar1 mRNA in the isolated choroid and isolated primary RPE (PRPE) cells; HCAR1 is exclusively expressed in the RPE layer. C: Confocal images of isolated PRPE staining with RPE65 (red); the nuclei of cells were counterstained with DAPI (blue). D: Visualization of vortex veins in the choroid from WT and Hcar1-KO (KO) pups at PT12 using podocalyxin (red) and α-smooth muscle actin (α-SMA; green). Four vortex veins are spotted at the equator of the choroid (white arrows). E: Top panels: Representative confocal images of the RPE layer from WT and Hcar1-KO (KO) mice at PT7, PT9, PT12, and PT15 stained with fluorescein isothiocyanate–phalloidin. Bottom panel: Histogram of average RPE cell area measured in the center of the eye cup. Data are presented as means ± SEM (E). n = 3 (A–C) to 4 (D) per group. Scale bars: 20 μm (C); 1 mm (D); 50 μm (E). [file mmc1.pdf]

**Fig S2**

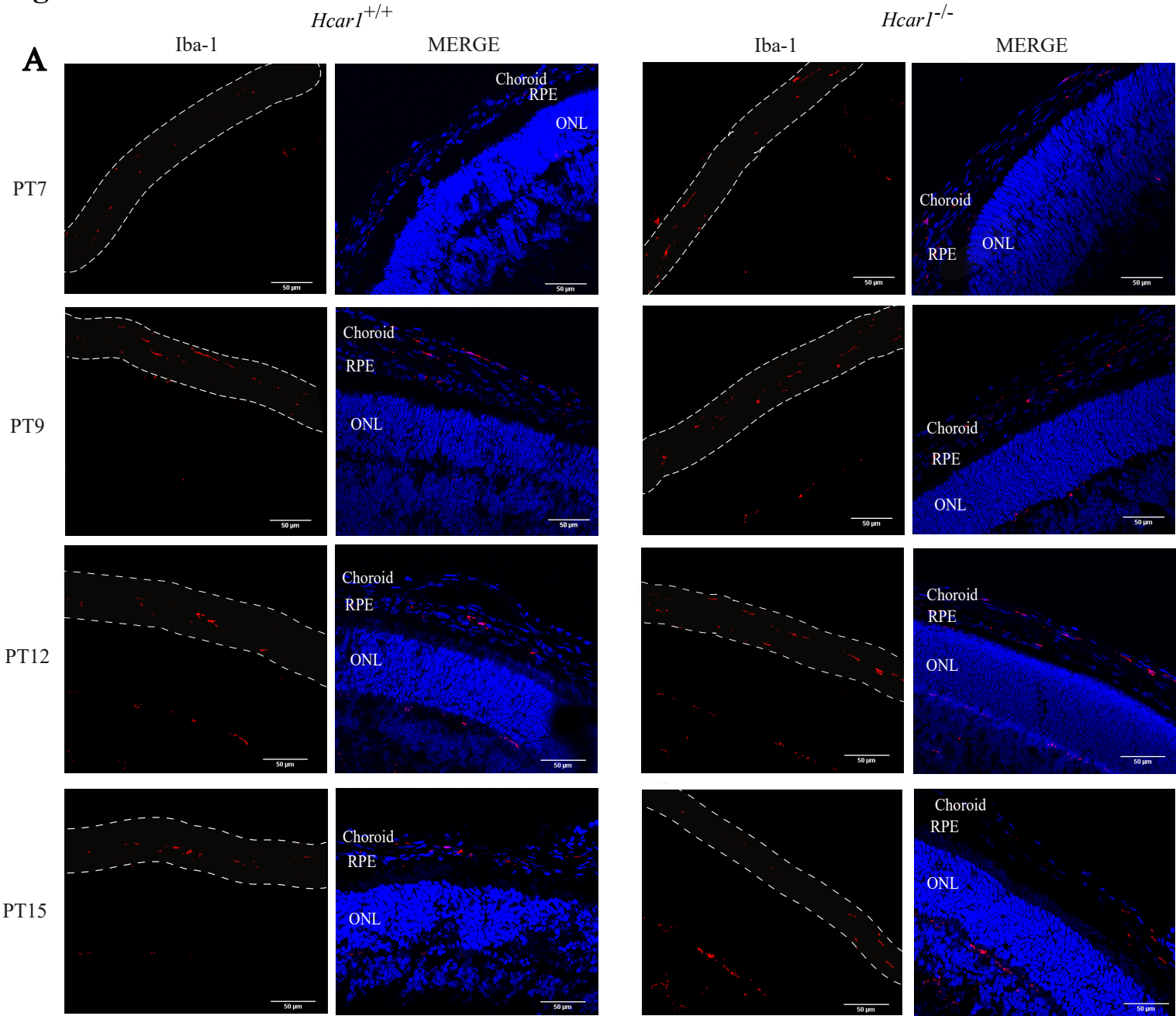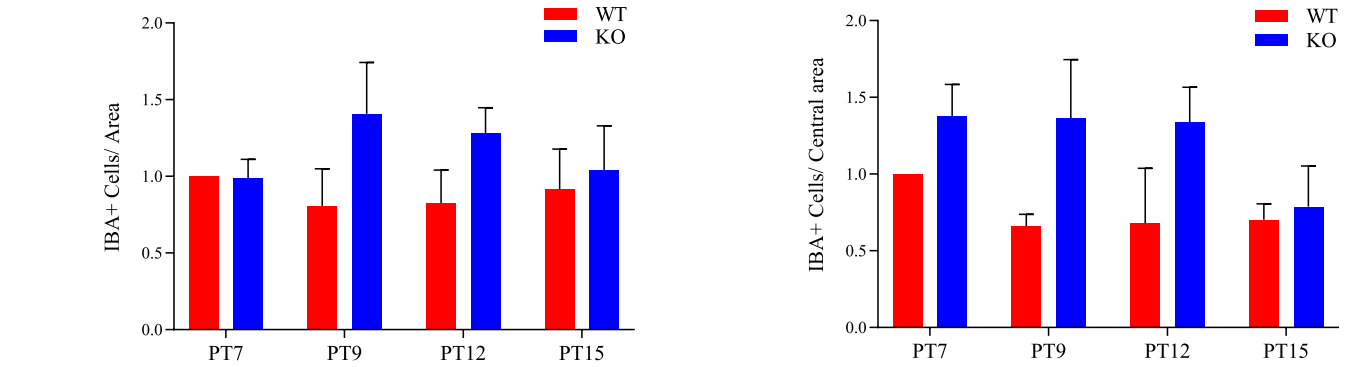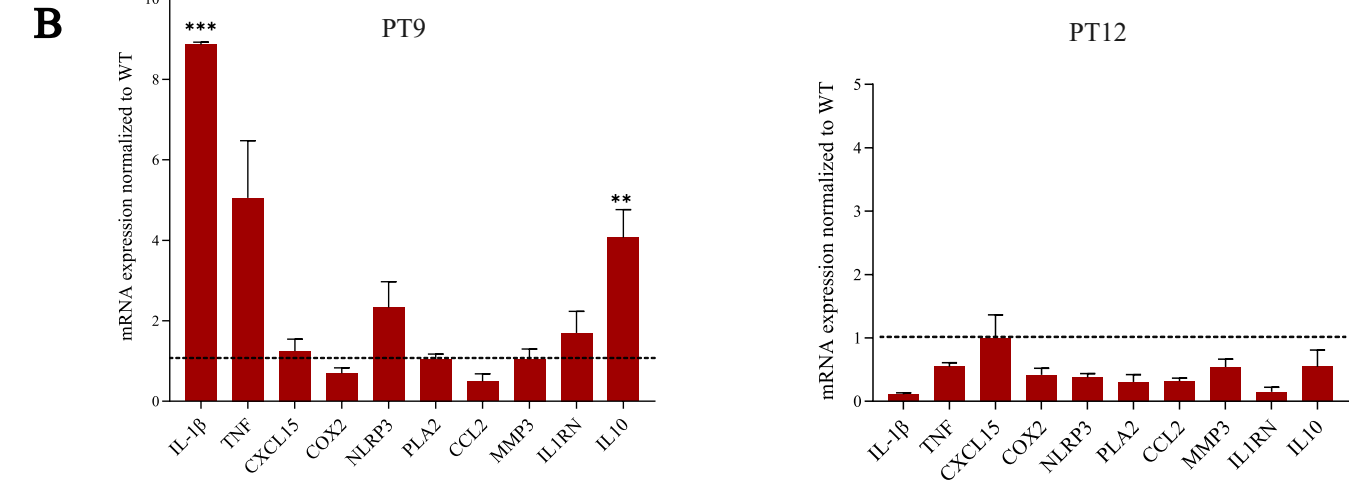

Supplement: Supplemental Figure S2 — Markers of inflammation in subretina of Hcar1-knockout (KO) and wild-type (WT) mice during development. A: Top panels:. Representative Iba-1 immunohistofluorescence (red) confocal images on cross-sections from WT and Hcar1-KO (KO) pups at PT7, PT9, PT12, and PT15; nuclei are counterstained with DAPI (blue). Subretina is delimited by a dashed line. Bottom panels: Quantification of Iba-1–positive cells in the subretina relative to the observed area, for each corresponding genotype and age and normalized to PT7 WT (left panel). Quantification of Iba-1–positive cells in the subretina relative to the central area, for each corresponding genotype and age and normalized to PT7 WT (right panel). B: mRNA levels of inflammatory factors measured by quantitative RT-PCR and presented as fold change in KO relative to WT animals, at PT9 (left panel) and PT12 (right panel). (The dotted line indicates the average gene expression in WT that was considered as 1.) Data are presented as means ± SEM (A and B). n = 3 to 6 per group. ∗∗P < 0.01, ∗∗∗P < 0.001. Scale bars = 50 μm (A). ONL, outer nuclear layer; RPE, retinal pigment epithelium. [file mmc2.pdf]

**Fig S3**

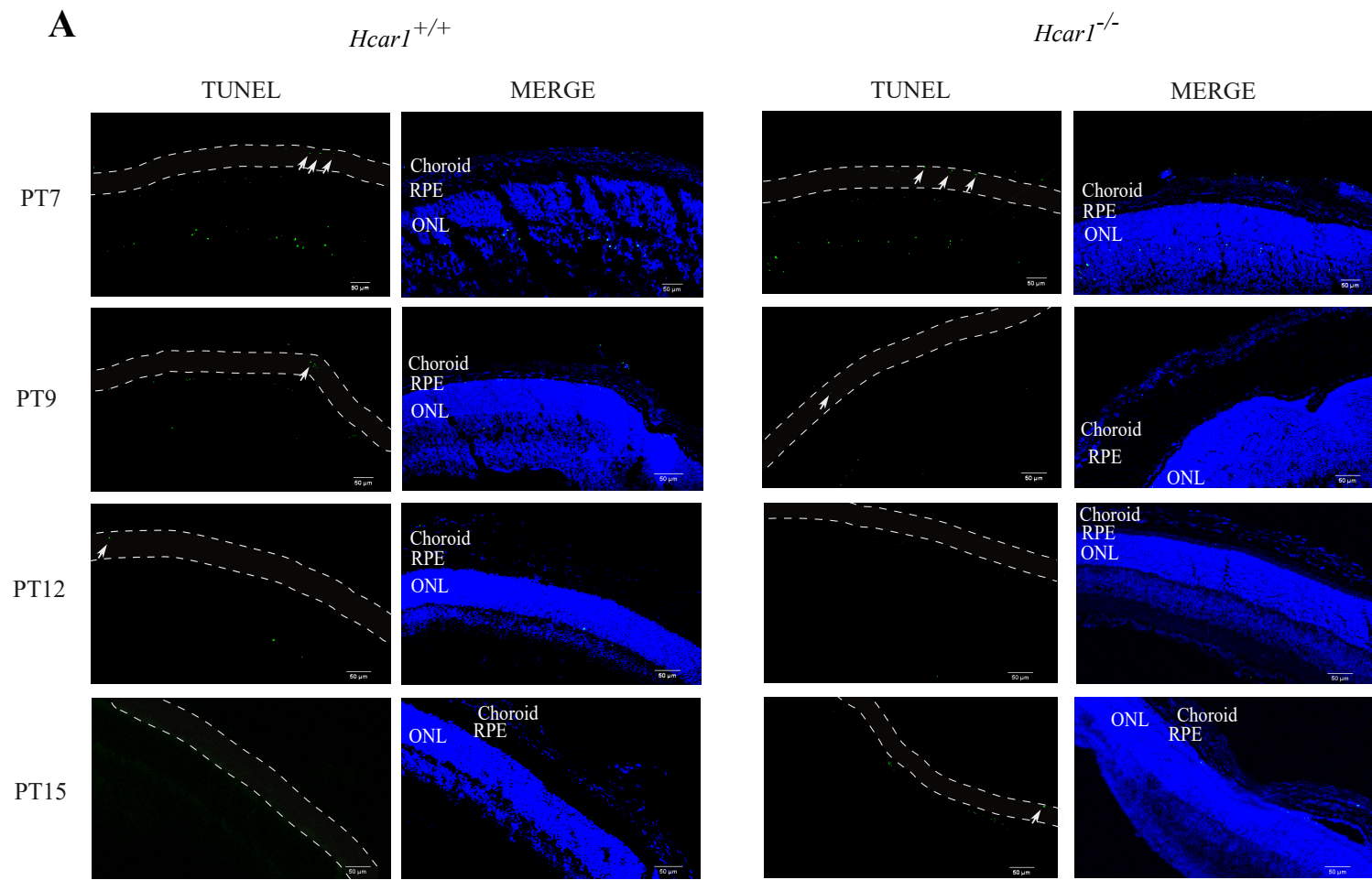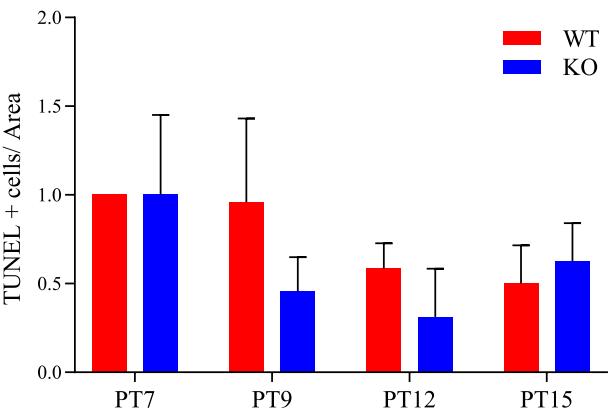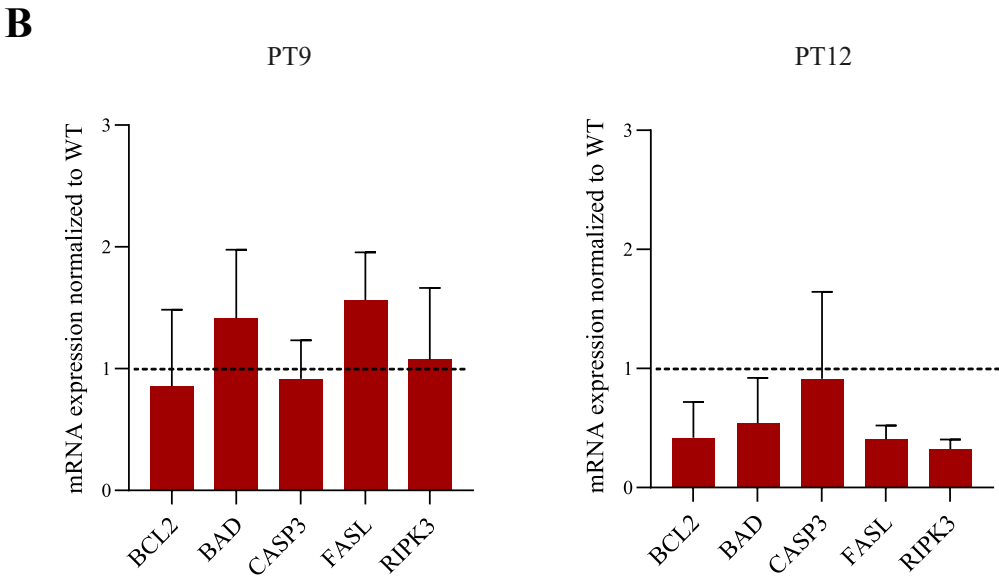

Supplement: Supplemental Figure S3 — Absence of an increase in apoptosis in subretina of young Hcar1-knockout (KO) mice. A: Top panels: Terminal deoxynucleotidyl transferase-mediated dUTP nick-end labeling (TUNEL) staining of subretina. Representative confocal images on cross-sections from wild-type (WT) and Hcar1-KO (KO) pups at PT7, PT9, PT12, and PT15; TUNEL-positive cells are shown in green and are highlighted with white arrows, and DAPI-stained nuclei are shown in blue. Subretina is delineated by a dashed line. Bottom panel: Quantification of TUNEL-positive cells in the subretina relative to the observed surface area, for each corresponding genotype and age and normalized to WT at PT7. B: mRNA levels of apoptotic factors measured by quantitative RT-PCR and presented as fold change in KO relative to WT animals, at PT9 (left panel) and PT12 (right panel). (The dotted line indicates the average gene expression in WT that was considered as 1.) Data are presented as means ± SEM (A and B). n = 3 to 6 per group. Scale bars = 50 μm (A). ONL, outer nuclear layer; RPE, retinal pigment epithelium. [file mmc3.pdf]

**Fig S4**

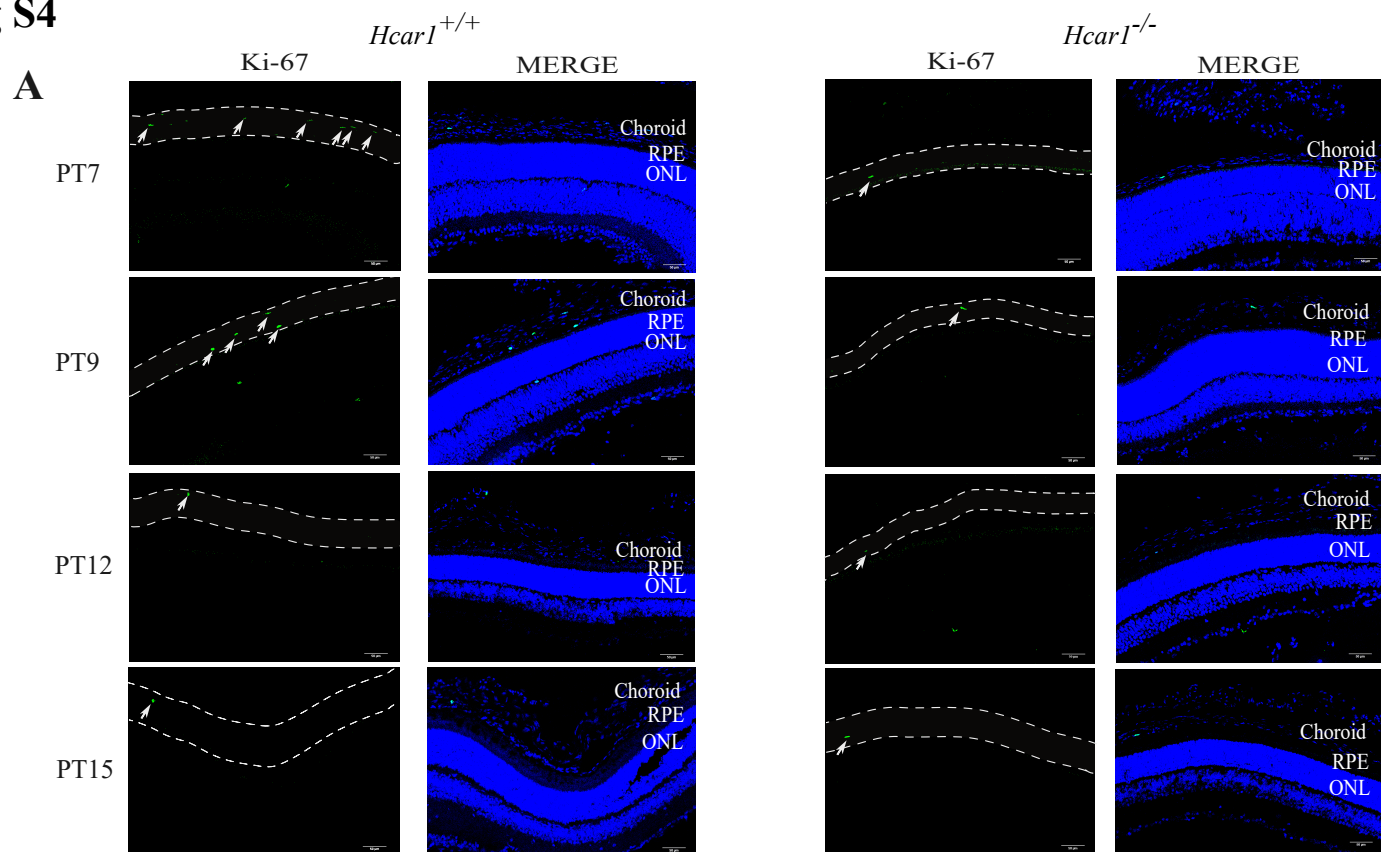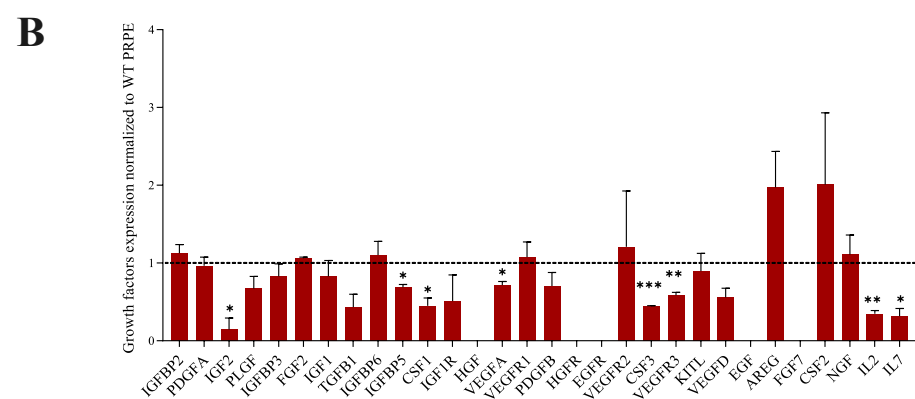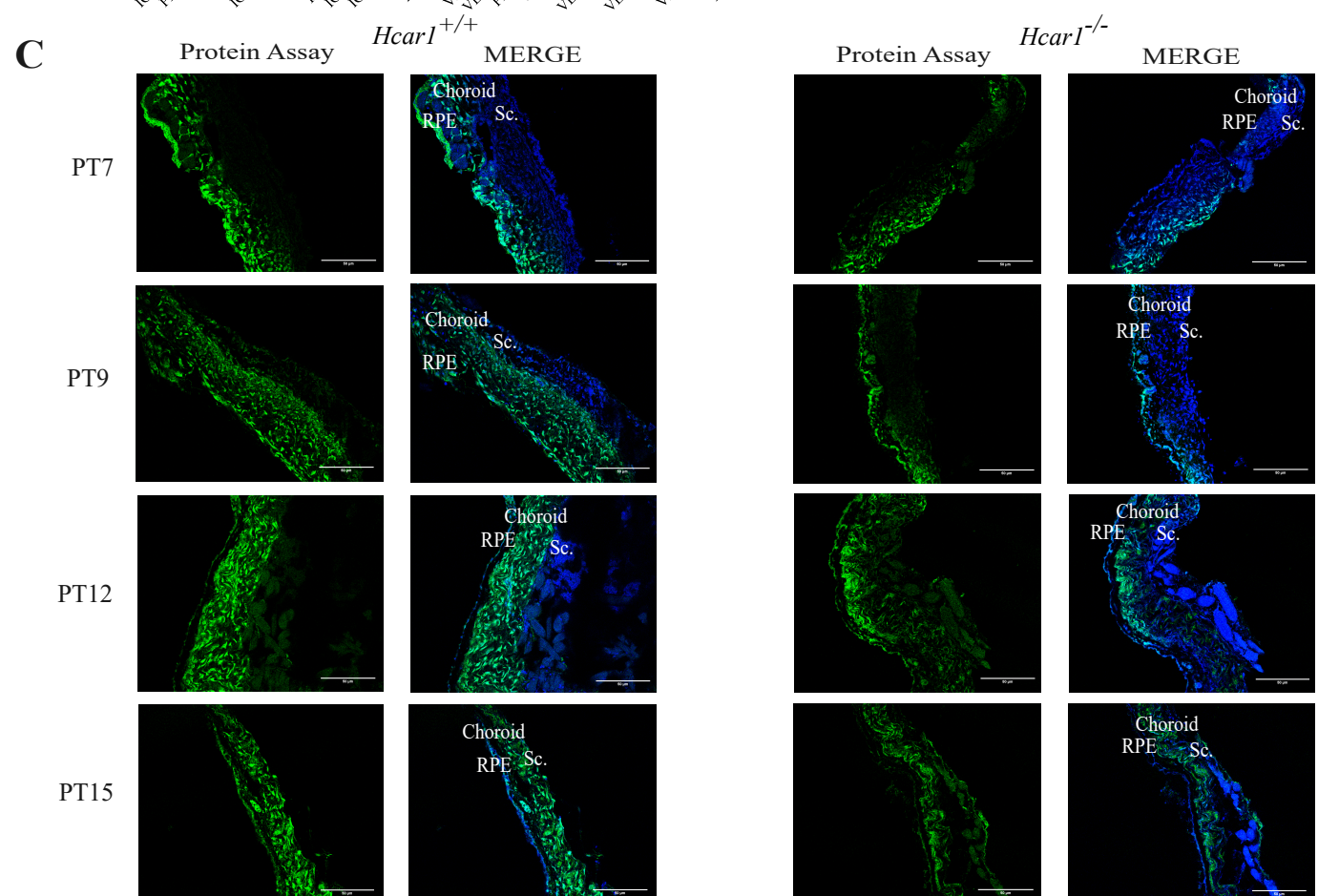

Supplement: Supplemental Figure S4 — Hcar1-knockout (KO) mice display a reduction in proliferation rate and protein synthesis. A: Representative confocal images of immunohistofluorescence of Ki-67 (green) on ocular cross-sections from wild-type (WT) and Hcar1-KO (KO) pups at PT7, PT9, PT12, and PT15; DAPI-stained nuclei are in blue. Subretina is delineated by a dashed line. White arrows point to single Ki-67–positive cells. B: Expression of growth factors was analyzed in primary retinal pigment epithelium (RPE) cells by Western blot analysis. Signal intensity from primary retinal pigment epithelium (pRPE) cells of KO mice was normalized to signal from pRPE of WT animals. (The dotted line indicates the average growth factors expression in WT that was considered as 1.) C: Representative confocal images of OPP-AZDye 488 incorporation (green); DNA was counterstained with Hoechst (blue). Data are presented as means ± SEM (B). n = 3 to 6 per group. ∗P < 0.05, ∗∗P < 0.01, and ∗∗∗P < 0.001. Scale bars = 50 μm (A and C). ONL, outer nuclear layer; Sc., sclera. [file mmc4.pdf]

Fig S5

A

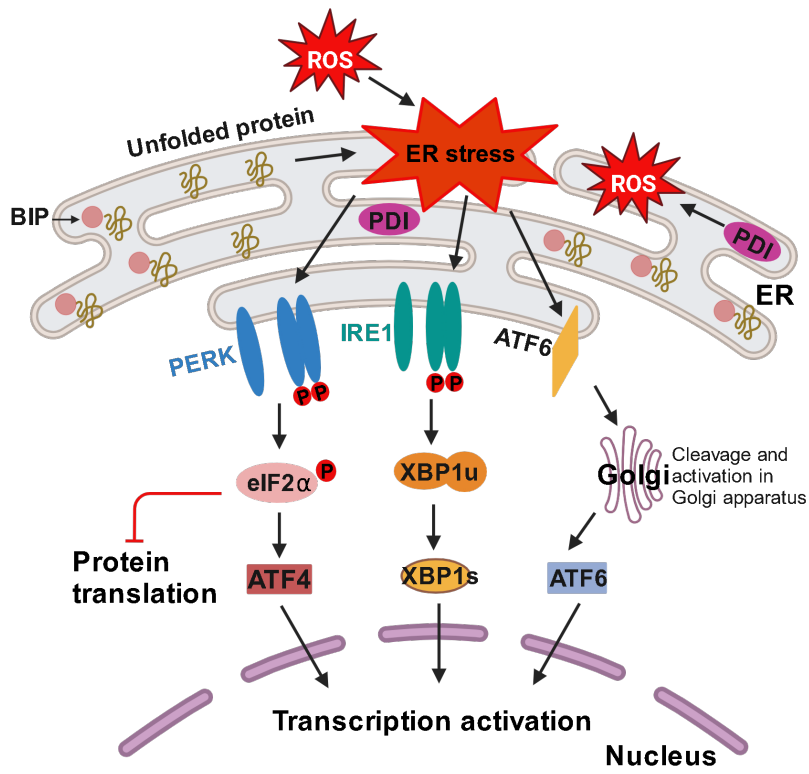

B

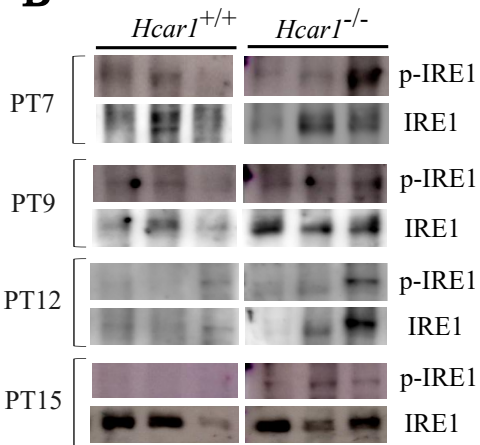

C

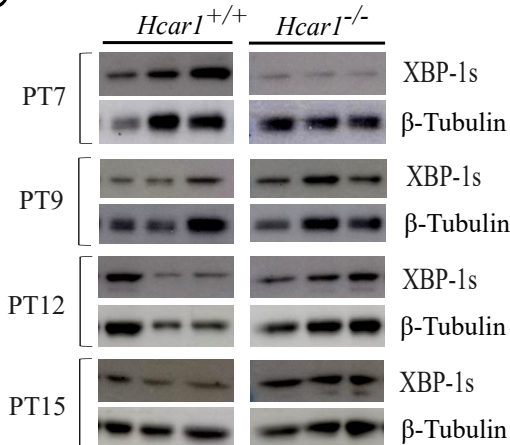

D

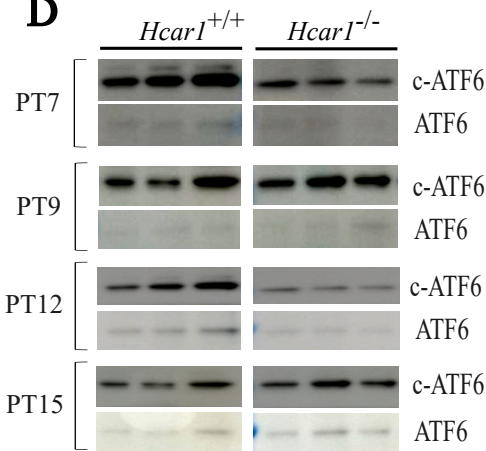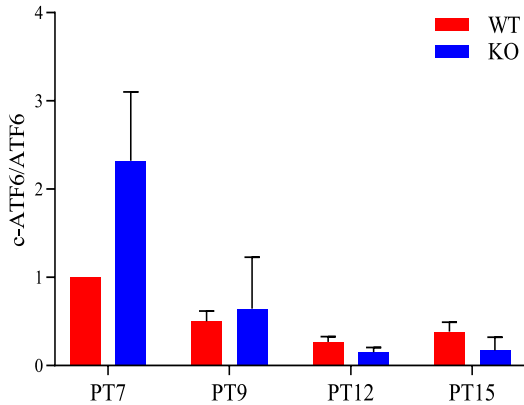

Supplement: Supplemental Figure S5 — Activation of unfolded protein response pathways in Hcar1-knockout (KO) mice. A: Schematic overview of the unfolded protein response pathway. Generated using BioRender.com (Toronto, ON, Canada). B: Representative Western blot analyses of phosphorylated inositol-requiring enzyme 1α (P-IRE-1α) compared with total IRE-1α in the subretina of wild-type (WT) and Hcar1−/− mice at PT7, PT9, PT12, and PT15. C: Representative Western blot analyses of spliced X-box binding protein-1 (XBP-1s) in the subretina of WT and Hcar1−/− mice at PT7, PT9, PT12, and PT15. D: Left panel: Representative Western blot analysis of cleaved activating transcription factor 6 (c-ATF6) in the subretina of WT and Hcar1−/− mice at PT7, PT9, PT12, and PT15. Right panel: Quantification of cleaved ATF-6 (c-ATF6) relative to unprocessed ATF6, in the subretina of WT and Hcar1−/− mice at PT7, PT9, PT12, and PT15. BiP, binding immunoglobulin protein; eIF2α, eukaryotic translation initiation factor 2α; ER, endoplasmic reticulum; PDI, protein disulfide isomerase; PERK, protein kinase RNA-like ER kinase; ROS, reactive oxygen species. [file mmc5.pdf]

**Fig S6**

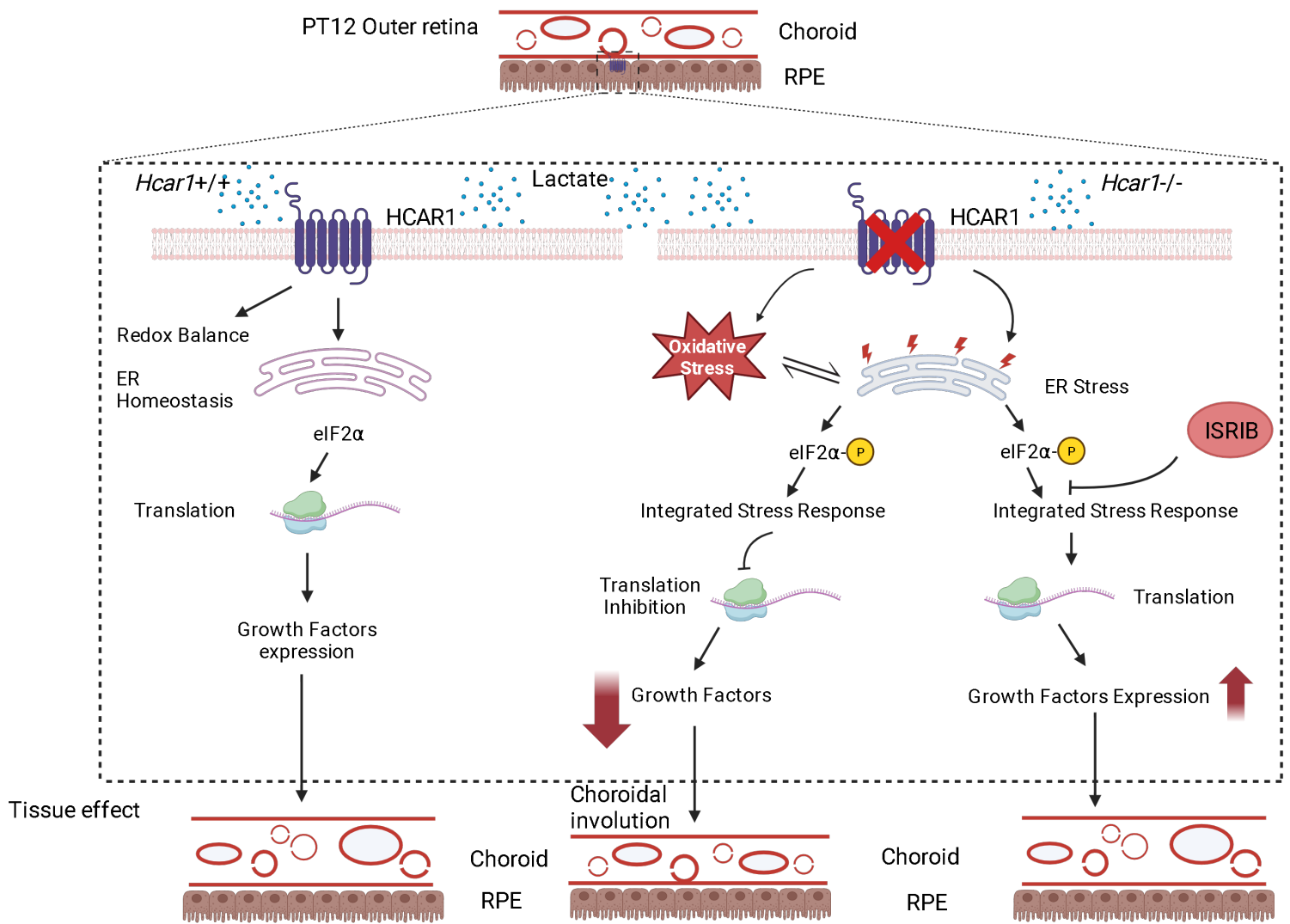

Supplement: Supplemental Figure S6 — Suggested model of the pathways affected by a lack of the Hcar1 gene. Proposed model depicting the pathways affected by the knockout of Hcar1 gene. HCAR1 deficiency leads to endoplasmic reticulum (ER) and oxidative stress, resulting in eukaryotic translation initiation factor 2α (eIF2α) phosphorylation and subsequent integrated stress response (ISR) pathway activation. ISR activation and consequent inhibition of protein translation leads to a decrease in growth factors expression, which, in turn, results in choroidal thinning. ISR inhibitor (ISRIB) rescues growth factor expression and choroidal thickness. Image generated using BioRender.com (Toronto, ON, Canada). RPE, retinal pigment epithelium. [file mmc6.pdf]
